# Threshold concepts in medical education: A scoping review

**DOI:** 10.1111/medu.14864

**Published:** 2022-07-24

**Authors:** Helen Jones, Lucy Hammond

**Affiliations:** ^1^ Warwick Medical School University of Warwick Coventry UK

## Abstract

**Introduction:**

The threshold concept framework (TCF) was first described nearly 20 years ago, but its application in the field of medical education has recently seen a significant growth of interest with a diverse range of literature published on the subject. The transformative nature of threshold concepts (TCs) offers potential for the design of learning experiences and curricula across the medical education continuum. A scoping review was conducted to map the extent of the current literature regarding TCs in medical education—to describe the types of available evidence and its focus—and identify research gaps.

**Methods:**

The review followed the JBI Manual for Evidence Synthesis approach for scoping reviews. Four databases and two additional websites were searched for articles exploring TCs in medical education. Data were analysed using quantitative and qualitative thematic approaches. A framework of conceptual change was used to synthesise the TCs identified.

**Results:**

Thirty‐six papers, spanning undergraduate, postgraduate and continuing medical education, were included in the final analysis. The most frequent application of the TCF was in the identification of TCs, which related to basic scientific knowledge, ways of thinking and ways of practising in medicine. Uncertainty, patient care, clinical reasoning and professional identify formation were themes that emerged at multiple stages of training. Several papers evaluated the use of the TCF in teaching.

**Conclusion:**

The understanding and embodiment of TCs increases in complexity across the medical education continuum, with TCs recurring with changes in clinical environment and responsibilities. This lends support to a holistic approach to curriculum design spanning all stages of training. Further research is needed to develop a consistent approach for describing and applying the TCF in medical education and to address how the TCF can be used in teaching and how threshold crossing can be measured.

## Abstract 2

Understanding and embodiment of threshold concepts across the medical education continuum is argued through this review to support a holistic approach to curriculum design.

## 1 BACKGROUND

Threshold concepts (TCs) were first described in 2003^1^ and are depicted as ‘akin to a portal opening up a new and previously inaccessible way of thinking about something’.^1^ ^(p1)^ Grasping a TC therefore enables transformation of the learner's understanding and perception of a subject or discipline which allows them to progress.^1^ Meyer and Land (2003) used *heat transfer* as an example: understanding that the temperature gradient determines the rate at which something cools, which transforms how someone thinks about cooking and may change what pots they use to best aid the process.^1^ As the threshold concept framework (TCF) has developed, several possible characteristics have been proposed, which students may experience to varying degrees. These are described in Table 1.

**TABLE 1:** Characteristics of a threshold concept^1^, ^2^, ^3^, ^4^

Transformative	Associated with a significant shift in how the learner views the subject or discipline.
Irreversible	Concepts are difficult to forget or unlearn.
Integrative	Grasping a concept reveals previously unseen relationships between discipline aspects.
Troublesome	Concepts appear counterintuitive or alien or are tacit in nature. They require students to redefine previously held knowledge and beliefs.
Bounded	Concepts are delineated within a specific context and have terminal frontiers.
Liminality	While engaging with the concept, the learner may oscillate between new and old understandings.
Reconstitutive	Crossing the threshold brings about a shift in learner subjectivity or identity.
Discursive	Crossing the threshold is associated with an elaboration of the learner's use of language.

TCs, therefore, represent more than basic building blocks of knowledge. They involve a journey towards mastery in which the learner occupies a liminal space while they grapple with new understandings.^2^, ^3^, ^5^ This struggle can present as mimicry and the learner may need support to successfully navigate this space.^2^, ^5^ Crossing a threshold subsequently leads to a change in ‘knowing, doing, being, and future learning possibilities’.^6^ ^(p263)^ This represents a transformative epistemological or ontological shift,^7^ enabling students to *think* in a discipline specific way, and is associated with a transfiguration of the learner's identity^1^, ^2^, ^3^, ^4^ as they *become* part of the discipline.^6^

TCs have therefore been utilised in health professions education to explore ways of thinking and practising.^6^, ^8^ In this context, the TCF provides a lens for exploring how curricula can best prepare students for real‐world practice and address issues which hinder professional identity formation.^8^, ^9^ In addition, it could aid students in integrating disjointed learning experiences^9^ and help educators focus on crucial concepts that open up greater understanding of the subject area^5^, ^9^—a useful tool in content‐heavy medical curricula. However, Brown et al. have challenged the suitability of the TCF in health professions curricula as it appears at odds with a constructivist paradigm by implying one body of knowledge and focusing on cognitive conceptions of identity.^10^ They argue TCs should not be used to structure curricular and instead as a reflective prompt to aid discussions between students and educators.^10^

There has been a growing interest in TCs in medical education in the last decade. While a qualitative synthesis of the health sciences TC literature was conducted in 2017,^8^ to the best of our knowledge, no systematic or scoping reviews have been conducted on the topic of TCs in medical education exclusively. Given the competing viewpoints, exploration of the literature is essential to establish the nature of the current discussion. It could also provide important insights to guide evidence‐based teaching practice and curriculum design. Therefore, the aim of this study is to explore and describe the current research regarding TCs across the medical education continuum (undergraduate, postgraduate and continuing medical education), consider how TCs are being used to inform practice in medical education, and to identify research gaps. Given the emerging and heterogeneous nature of the evidence, a scoping review was selected to map the extent of the current literature.^11^

## 2 METHODS

The review followed the framework presented in the JBI Manual for Evidence Synthesis^12^ and adhered to Preferred Reporting Items for Systematic reviews and Meta‐Analyses extension for Scoping Reviews (PRISMA‐ScR).^13^ The PRISMA‐ScR checklist can be found in Appendix S1. The research questions, study selection criteria and methods were published in a protocol.^14^

### 2.1 Search strategy

Medline (Ovid), Embase (Ovid), Scopus (Elsevier) and Education Research Complete (EBSCO) were searched on 29 July 2021. Minor changes were made to the search strategy presented in the protocol: Line 8 of the of the original strategy was removed as it was too broad, and ‘liminal’ and ‘transformative’ were truncated to capture iterations of the terms. The search was limited to papers published since 2003, when the TCF was first described,^1^ and to papers in the English language. The Medline search strategy is shown in Table S1.

Additional papers were identified from the online Threshold Concepts Bibliography which lists a range of publications from 2003 to 2018.^15^ Hand‐searching of MedEdPublish, an open access platform with a post‐publication peer review process,^16^ was undertaken to identify grey literature. The reference lists of the articles included for full text review were also hand‐searched.

### 2.2 Screening articles

The references for the identified papers were uploaded to Covidence.^17^ Following de‐duplication, authors HJ and LH independently screened the title and abstracts against the study selection criteria. An inclusive approach was taken, and abstracts that referenced health professions were included to establish whether relevant data could be extracted from the full text. Full texts of the selected articles were then reviewed against the inclusion and exclusion criteria independently, to obtain a final selection of papers. We met at the beginning, midpoint and end of the screening process and resolved any disagreements about study inclusion via discussion.

### 2.3 Study selection criteria

The inclusion and exclusion criteria were developed using the population, concept, context (PCC) format^12^ (Table 2). To ensure that a thorough picture of current thinking was obtained, all types of publications were included. Abstracts without an accompanying paper were excluded. A taxonomy developed by Barradell and Peseta in the context of health professions education^8^ was incorporated into the inclusion criteria to aid charting and analysis.

**TABLE 2:** Study selection criteria

Inclusion criteria
Population ○ Undergraduate/graduate‐entry medical students ○ Postgraduate medical trainees/residents/physicians ○ Medical educators
Concept ○ Threshold concepts ○ Fulfils at least one aspect of the taxonomy developed by Barradell & Peseta^8^
Context ○ Undergraduate, postgraduate or continuing medical education
Types of study to be included ○ All types of paper, including empirical studies, editorials, perspective and opinion pieces and reviews
Exclusion criteria
○ Studies involving other health care professions or health sciences, where: Medical trainees/physicians/medical educators are not included Or it is not possible to identify and extract data specifically related to medical trainees/physicians/medical educators ○ Studies not in English language ○ Literature which consists of an abstract only with no accompanying paper, e.g., conference abstracts

### 2.4 Data charting

A data charting form was developed and piloted prior to publication of the protocol.^14^ The form aimed to capture key details relevant to the review questions,^12^ including the relationship to the taxonomy and our findings.^8^ HJ charted the data for the included studies, and LH verified it for accuracy. Any disagreements were resolved by discussion. It became apparent that some authors used different terminology, such as threshold skills and vocational thresholds, and the decision was made to apply the taxonomy to these papers in a comparable way to ensure no data loss.

### 2.5 Analysis

We used a descriptive quantitative analysis to examine key details about the context and approach of the papers, to present a numerical representation of the extent of the literature and to provide context for the qualitative synthesis.

Qualitative analysis followed an inductive approach and codes were developed from the extracted data. The coding was an iterative process and involved reanalysing the papers as new findings emerged. We subsequently grouped similar codes to identify themes within a framework of conceptual change, developed by Davies and Mangan in their work in economics.^18^ Barradell and Peseta described the applicability of this framework to TCs associated with the development of professional thinking and practice in physiotherapy education^6^:

- *Basic conceptual change* involves reforming knowledge in relation to its meaning and language within the discipline.^18^ In medical education, this includes basic scientific and medical knowledge.
- *Discipline conceptual change* involves learners using the knowledge they have acquired and understanding the relationships between concepts.^18^ This is likened to ways of thinking within the medical discipline.^6^
- *Modelling conceptual change* encompasses reasoning and the construction of arguments that are rooted in the discipline.^18^ This is akin to ways of practicing as a physician.^6^

The qualitative analysis was rooted in subjective epistemology and represents a synthesis of the evidence based on author interpretation.^19^ Consistent with a scoping review approach, a formal review of the quality or risk of bias of the included papers was not undertaken.^20^ However, a critical lens was applied during data charting, and we considered the experimental rigour of the included studies. This is incorporated under ‘Our findings’ in Appendix S2 and informed the focus of discussion and subsequent recommendations.

## 3 RESULTS

### 3.1 Summary of included studies

**FIGURE 1:**
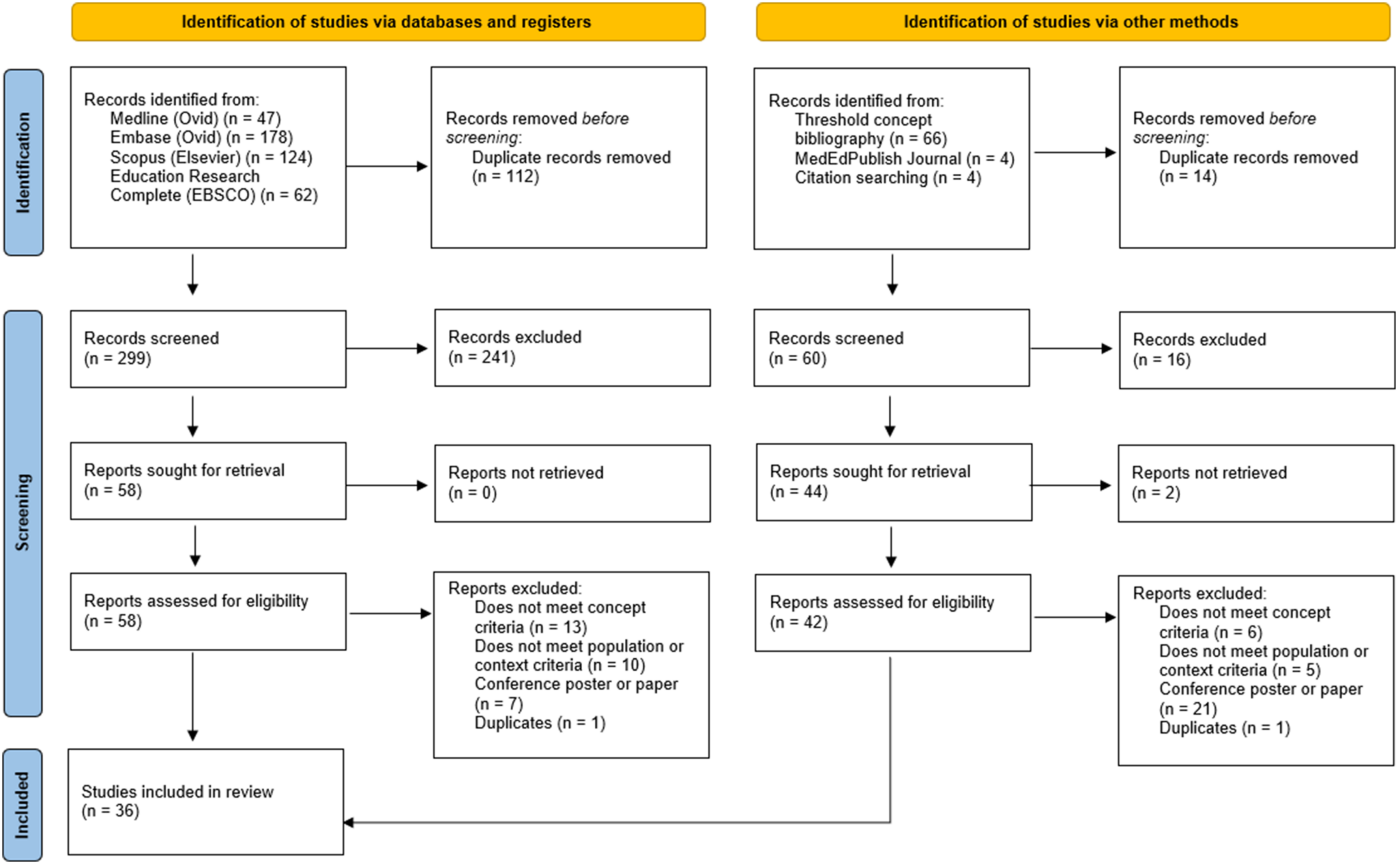
PRISMA flow diagram^56^ [Color figure can be viewed at wileyonlinelibrary.com]

Thirty‐six papers published between 2012 and 2021 were included in the final analysis (see Figure 1 for PRISMA flow diagram). Most papers originated in the United Kingdom (*n* = 15^21^, ^22^, ^23^, ^24^, ^25^, ^26^, ^27^, ^28^, ^29^, ^30^, ^31^, ^32^, ^33^, ^34^, ^35^), North America (*n* = 11^36^, ^37^, ^38^, ^39^, ^40^, ^41^, ^42^, ^43^, ^44^, ^45^, ^46^) and Australasia (*n* = 7^47^, ^48^, ^49^, ^50^, ^51^, ^52^, ^53^). Some papers focused on particular medical specialities and subject areas, including surgery (*n* = 5^21^, ^23^, ^41^, ^47^, ^54^), medical physiology and biomedical science (*n* = 4^42^, ^43^, ^51^, ^55^), geriatric medicine and dementia care (*n* = 3^26^, ^27^, ^29^), psychiatry (*n* = 2^24^, ^35^), general practice (*n* = 2^34^, ^50^), military medicine (*n* = 2^36^, ^37^), anaesthetics,^46^ internal medicine,^39^ neurology,^40^ paediatrics,^38^ palliative care,^48^ population health,^28^ rheumatology^52^ and transplant science.^30^ A wide range of article types were identified (Table 3), the majority of which were qualitative research studies. A detailed summary of the papers is included in the Appendix S2.

**TABLE 3:** Types of journal articles and research designs in the review

Type of article and research design	Number in review
Primary research studies	
Qualitative study	16^21^, ^22^, ^23^, ^24^, ^25^, ^26^, ^27^, ^36^, ^37^, ^38^, ^39^, ^40^, ^47^, ^48^, ^49^, ^50^
○ Grounded theory study	5^21^, ^24^, ^36^, ^38^, ^39^
○ Phenomenological study	2^23^, ^26^
○ Ethnographic study	1^22^
○ Naturalistic enquiry	1^25^
○ No specific research design stated	7^27^, ^37^, ^40^, ^47^, ^48^, ^49^, ^50^
Mixed method study	6^28^, ^29^, ^41^, ^51^, ^54^, ^55^
Quantitative study	2^30^, ^52^
Mixed (conceptual analysis and qualitative study)	1^31^
Other publications	
Commentary	4^42^, ^43^, ^44^, ^45^
Conceptual analysis	3^32^, ^33^, ^46^
Narrative review	1^53^
Theoretical paper	1^57^
Comment	1^34^
Letter to the editor	1^35^

### 3.2 TCs identified

The most frequent application of the TCF in medical education was in the identification of TCs. Ten papers focused on identifying TCs in undergraduate medical education.^22^, ^24^, ^25^, ^28^, ^36^, ^37^, ^38^, ^42^, ^49^, ^53^ Four papers examined TCs in junior postgraduate medical education (encompassing the roles in different health care systems of interns, foundation year doctors or core trainees) or senior postgraduate medical education (senior residents, specialist trainees, registrars or fellows).^21^, ^23^, ^26^, ^48^ One paper looked at both undergraduate and junior postgraduate medical education,^39^ and one paper focused on continuing medical education involving senior clinicians.^47^

A variety of data collection methods were used by the primary researchers to identify potential TCs including interviews,^21^, ^23^, ^24^, ^26^, ^39^, ^40^, ^47^, ^49^ focus groups and discussion,^25^, ^28^, ^42^, ^48^ written reflections,^36^, ^37^, ^38^ audio‐diary recordings^22^, ^25^ and questionnaires.^26^, ^28^ Data were gathered from the population of interest,^21^, ^22^, ^36^, ^37^, ^38^, ^42^, ^47^, ^48^, ^49^ educators or experts^28^, ^40^ or from both learners and trainers to triangulate the data around possible TCs.^23^, ^24^, ^25^, ^26^, ^39^

### 3.3 Basic conceptual change

Only two papers used empirical methods to examine TCs in this area.^40^, ^42^ Potential TCs were identified in relation to physiology and biomedical science, including secondary messenger systems, pressure gradients, preload and afterload, cell membrane potential and polarity.^40^, ^42^ Ventilation‐perfusion (V/Q) mismatch^46^ and an X‐ray being a 2D representation of a 3D structure^32^ were proposed as TCs based on professional experience. Evaluation of this type of TC is of fundamental importance because grasping them may help in the subsequent mastery of other difficult concepts and development of skills.^40^

### 3.4 Ways of thinking (discipline conceptual change)

Three themes emerged relating to ways of thinking within a discipline: uncertainty, care of patients and the doctor's role and development of professional identity.

Recognising that uncertainty is an inherent part of medicine emerged in several papers.^22^, ^25^, ^28^, ^38^, ^39^ Closely related was the realisation that medicine is not black and white.^22^, ^38^ Crossing these thresholds led to an understanding that there was ‘no single solution’^25^ ^(p100)^ in the diagnosis and management of patients. After initially struggling with ambiguity, however, ‘students became comfortable with holding multiple possibilities simultaneously in their heads’.^37^ ^(p7)^ They recognised that the reality of medical practice is more complex than the textbook picture and that learning is lifelong and it is not necessary to know everything.^25^, ^38^

Person‐centred care as a TC was described in context of seeing the patient as a whole^26^, ^39^ and that there was ‘more to the practice of medicine than just the clinical application of biomedical science’.^25^ ^(p98)^ This led to a transformation in how students saw health.^25^ Recognising the importance of exploring patients' perspectives and goals was also noted to be an essential feature of patient‐centredness.^39^ In addition, the doctor's role in the multidisciplinary team^26^ and the importance of collaboration with other health professionals^39^ was seen as key in providing effective care and transformed how trainees viewed the role of others in the team.

The development of professional identity was common feature of several TCs in the review.^25^, ^28^, ^36^, ^37^, ^38^, ^39^, ^47^ There was a realisation that ‘I am a healer’^37^ ^(p16)^ and an awareness of the professional culture students were entering into.^25^ This transformation was uncomfortable and challenged students' personal identity^25^ as they recognised their responsibility for others' health.^37^ The formation of a unique professional identity was noted in the context of military medicine.^36^, ^37^ However, there was also a recognition of the multiple wider roles that doctors play^25^, ^39^ and that their responsibility extends beyond the individual patient.^28^

### 3.5 Ways of practising (modelling conceptual change)

Four themes emerged relating to ways of practising: development of practical skills, clinical judgement and decision making, tolerance of uncertainty and enactment of patient‐centredness and the provision of holistic care.

Practising as a clinician involves the development of unique practical skills. Technical complexity and speed of operating were identified as TCs amongst qualified surgeons.^47^ However, non‐technical factors such as team dynamics can influence procedural complexity,^47^ suggesting other TCs may underlie surgical management. Similarly, a key factor in why surgical trainees found procedures troublesome was lack of opportunities to practice.^21^, ^23^, ^41^ It has therefore been argued that some skills can be learnt with sufficient time and practice and so do not represent TCs.^23^, ^45^

In contrast, Evgeniou et al. identified potential TCs related to decision making during the pre‐operative, intra‐operative and post‐operative stages of a patient's treatment.^23^ Clinical reasoning was also recognised as a threshold skill in medical students, transforming how they gathered and analysed information.^49^ The ability to tolerate and respond appropriately to uncertainty is an underlying feature, and a possible TC, in the development of clinical judgement and decision‐making skills^21^, ^47^ and changed how students approached diagnoses.^24^ Similarly, uncertainty about illness trajectory and life expectancy shaped trainees' development of new ways of communicating with patients.^48^

Finally, several papers identified TCs related to patient‐centredness and holistic care,^24^, ^26^, ^37^, ^48^ which differed from discipline conceptual change, as ‘lip‐service’^48^ ^(p425)^ was transformed to the embodiment of these principles in professional practice. Considering psychosocial aspects in the assessment and treatment of patients was a key aspect of this theme.^24^, ^26^ In addition, the development of empathy and the ability to manage emotions changed how trainees interacted with patients.^25^, ^26^, ^48^ In geriatrics, providing care was seen as an active, hands‐on process, and as trainees *become* geriatricians, they embody the concepts of a nurturing practitioner.^26^

### 3.6 Relationship between TCs identified and stage of training

**FIGURE 2:**
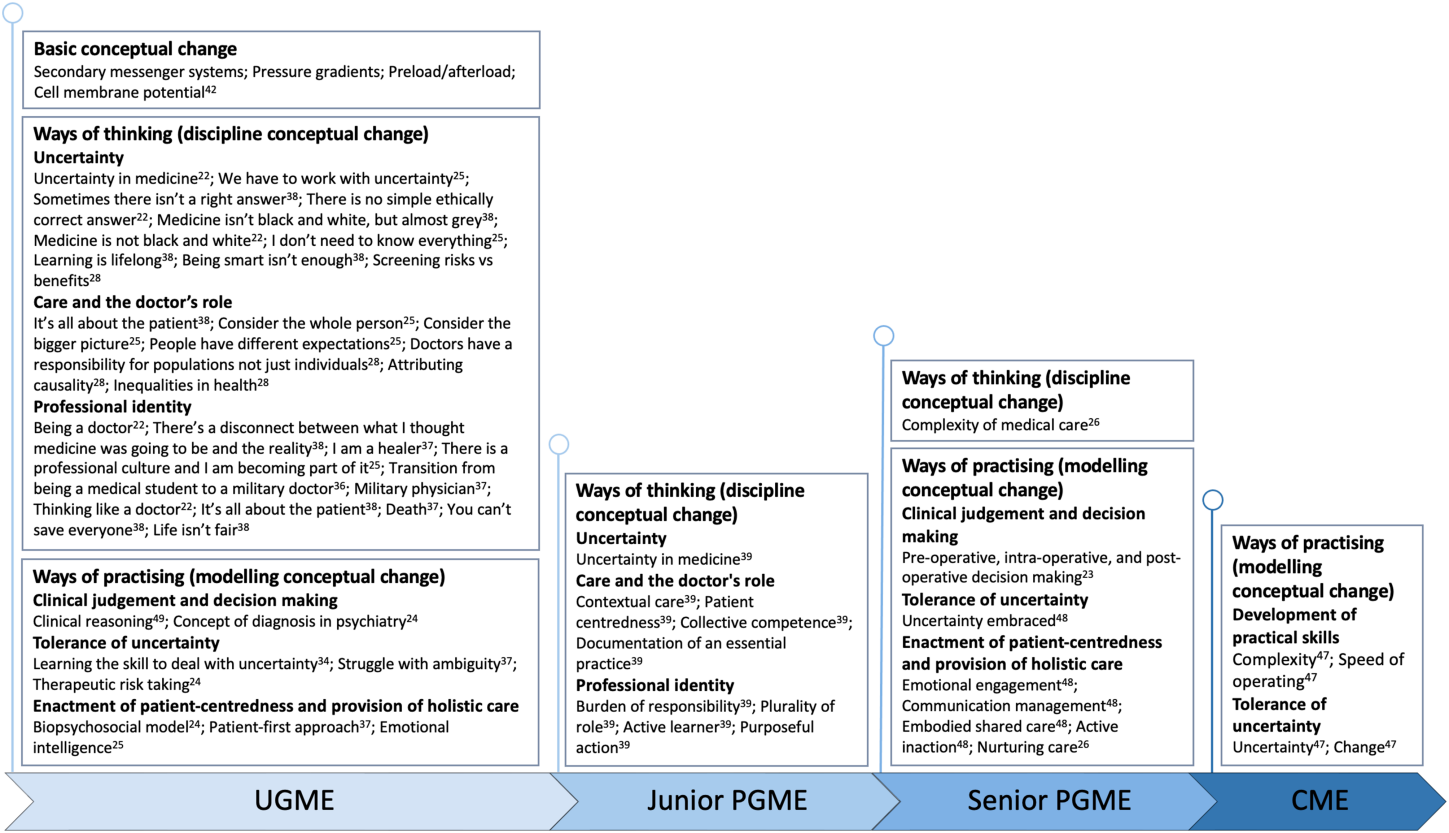
Threshold concepts identified at different stages of training: undergraduate medical education (UGME), junior postgraduate medical education (PGME), senior postgraduate medical education (PGME) and continuing medical education (CME) [Color figure can be viewed at wileyonlinelibrary.com]

The types of conceptual changes learners encounter appeared to relate to the stage of medical training, as shown in Figure 2. TCs related to scientific knowledge were identified in medical students, whereas TCs related ways of practising spanned the medical education continuum. Some TCs, such as uncertainty and patient care, featured at all stages of training, but developed in relation to professional responsibilities. There was also a recognition that as medicine advances TCs also evolve.^54^

### 3.7 Use of the TCs framework in teaching

Several papers evaluated teaching interventions in the context of the TCF^41^, ^43^, ^51^, ^54^, ^55^ and one paper incorporated the TCF into curriculum design of a new course.^30^ Other authors took a different approach and examined learning experiences associated with threshold crossing.^27^, ^50^ Students demonstrated a change in language, knowledge and behaviour following a simulation session about dementia care, although the TCs students may have crossed were not discussed.^27^ By examining written accounts of powerful learning experiences during GPs' development, Vaughan identified the importance of interactions with patients and colleagues in transforming trainees' dispositional attitudes.^50^

Many other papers suggested methods to teach the TCs they had identified. It was proposed that simulation could aid transformation in ways that are directly related to clinical practice,^46^ for example, by helping learners manage uncertainty.^47^ Alternatively, small reflective groups and Balint groups could help students navigate the challenges associated with professional identity formation.^22^, ^24^, ^25^ Strategies that trainers could use to support individual learners included discussion of cases and challenging situations,^23^, ^39^, ^45^ explicit diagnostic reasoning,^24^, ^49^ and formal mentoring programmes.^21^, ^47^ Authors also advocated for actively engaging students with the TCF,^22^, ^28^, ^33^, ^38^ enabling them to identifying their own TCs,^32^, ^42^ and recognise the repetitive, non‐linear and potentially troublesome learning process.^40^, ^45^ At a course level, several authors argued for the incorporation of TCs into curriculum design.^23^, ^25^, ^28^, ^47^, ^48^

## 4 DISCUSSION

This is the first review to take a systematic approach in examining the current TCF literature in medical education. Over two thirds of the papers included were published from 2018 onwards, demonstrating a significant growth in interest in recent years. This review is therefore highly pertinent in the current educational landscape and brings together a diverse evidence base.

### 4.1 Recursive TCs and implications for curriculum design

This synthesis has revealed a unique perspective on the recursive nature of TCs across the medical education continuum. Recursion was described by Land et al. (2005) as a revisiting of learning as students journey through the liminal space ‘attempting different “takes” on the conceptual material’.^3^ ^(p59)^ The medical education continuum is likewise an ongoing learning journey, in which learners revisit learning and adapt or advance it when faced with new situations.^48^ Uncertainty, for example, was a concept that featured at all stages of training and professional development, linked to both discipline conceptual change^22^, ^25^, ^28^, ^38^, ^39^ and modelling conceptual change,^21^, ^24^, ^34^, ^47^, ^48^ and re‐emerged with new roles and responsibilities. Medical students and junior postgraduate trainees first recognised the complex and ambiguous nature of medicine.^25^, ^38^ They then began to incorporate this into their decision making^24^, ^34^ and take responsibility for patients' care despite uncertainty.^25^, ^38^ However, senior clinicians also experienced uncertainty following their transition to independent specialty practice, related to decision‐making, clinical judgement and managing adverse events.^47^

**FIGURE 3:**
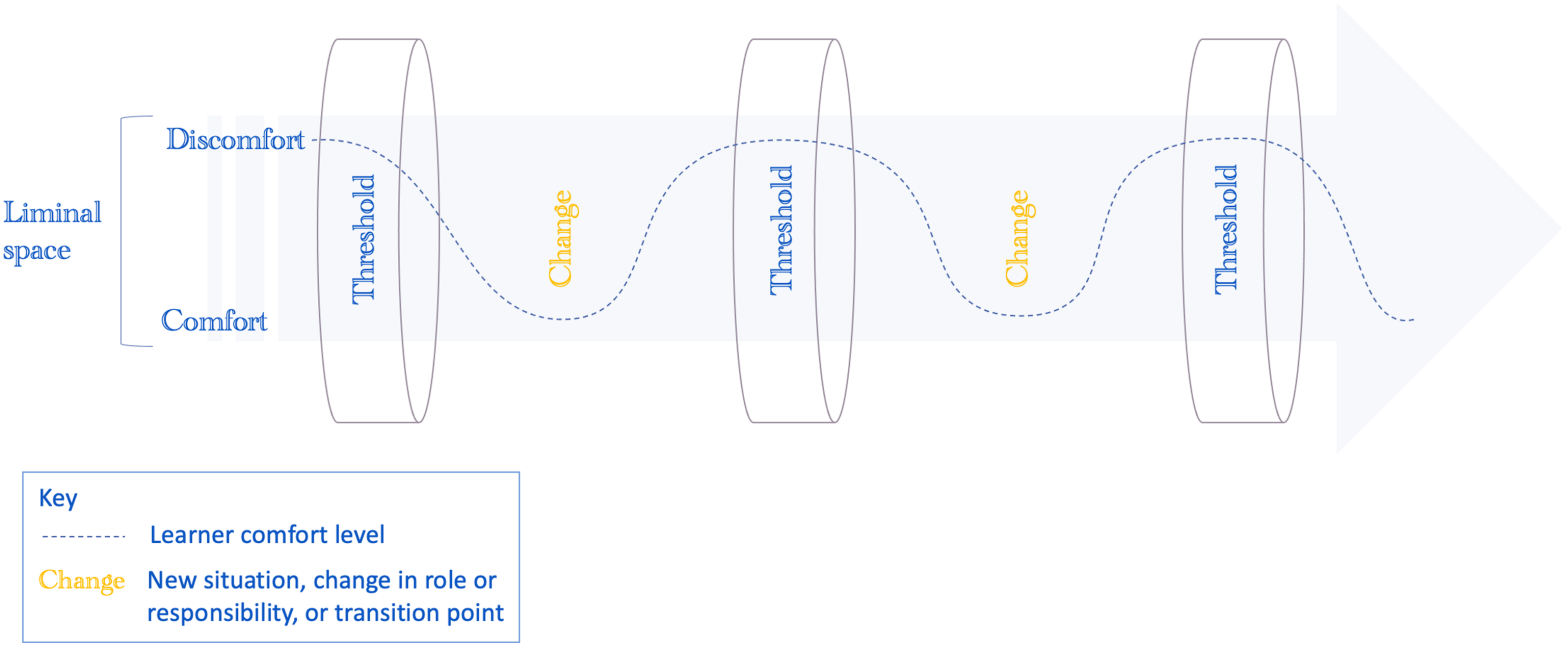
The recursiveness of threshold concepts and the ongoing liminal space in the medical education continuum [Color figure can be viewed at wileyonlinelibrary.com]

TCs themselves may therefore recur at various points in a professional's career, requiring a more sophisticated understanding or embodiment. This supports the notion that crossing a threshold is not the endpoint of the learning journey and that liminality may be better thought of as a state continually occupied by professionals, but which becomes less comfortable with exposure to new situations.^4^, ^47^ This is demonstrated in Figure 3. Viewing TCs in this way is a valuable insight which could help guide curricula design. It supports a holistic approach that spans the medical education continuum, as it has been argued that better integration between different stages of training would improve continuity in learning and help trainees navigate challenging transitions.^58^

The recursive nature of TCs would also, on face value, seem to support the spiral curriculum approach employed by many medical schools,^59^ in which topics are revisited throughout the course with increasing complexity and requiring more advanced application.^60^ However, this approach suggests a linear and progressive process, in which new learning builds on previous learning.^59^, ^60^ TC acquisition, in contrast, does not follow a predictably spaced process: It is a messy and back‐and‐forth journey, with deviations and revisions of understanding and outcomes.^3^, ^5^ Furthermore, the learning experience associated with TCs is unique to individuals and learners may pass through them at different points in their training.^26^, ^39^, ^50^ As a result, the spiral curriculum may not align with their learning needs, and they may progress in training without having crossed certain thresholds.^25^, ^39^

In contrast, the conceptualisation of the TCF in this review aligns more closely with competency‐based medical education (CBME), which is increasingly being applied in postgraduate medical education.^61^ In CBME, progression of the learner is determined by achieving competencies related to practicing medicine instead of time in training,^61^ often measured practically as Entrustable Professional Activities (EPAs) which are tasks or responsibilities trainees can undertake without supervision.^62^ Many of the TCs identified in this review represent ontological transformations and the development of professional practice, with learning rooted in clinical experience. The TCF could therefore be used as a lens for identifying these critical competencies or EPAs^39^ and help differentiate between mimicry and mastery.^46^ The recursive nature of TCs also supports the vertical integration of CBME which aims to ensure coherence across the medical education continuum.^61^ Aligning the TCF with CBME addresses some of the concerns raised by Brown et al. with regard to the promotion of a single body of knowledge and perpetuation of traditional power imbalances in education.^10^ A key component of CBME is a learner‐centred approach^61^ which can account for the individual variation in the experience of TCs. Indeed, Brown et al. argue that EPAs are a preferable component of curriculum design to TCs,^10^ but we contend that the two do not necessarily need to be viewed exclusively.

### 4.2 The meaning of threshold ‘concepts’ in medical education

This review also helps to address some of the criticisms around the use of the term threshold *concept* in medical education. As many authors have noted, the term concept has connotations of a theoretical, rather than practical, dimension^6^, ^10^ and so is often associated with ‘a content‐focused view of knowledge’.^4^ ^(p235)^ In this vein, Brown et al. have argued that the TCF is not applicable to professional identity development due to the focus on cognitive transformation—a change in knowledge leading to change in identity—and the disconnection between some TCs, such as those related to basic scientific knowledge, and professional identity.^10^ However, by viewing TCs in the context of stages of conceptual change, this review clarifies how crossing thresholds leads to changes in ways of thinking and practising within the discipline of medicine, and this is intrinsically linked to reshaping of a learner's identity.^18^

This knowledge‐focused interpretation of the TCF has also led to the emergence of alternative terminology. Two studies identified in this review used the terms threshold skills^49^ and vocational thresholds,^50^ respectively. The justification was that these ideas represent *how* knowledge is acquired and utilised^49^ and represent transformations related to *being* a practitioner.^50^, ^63^ However, the original description of TCs was broad and inclusive of values, attitudes, skills and ways of thinking and being.^1^, ^2^, ^4^ This explanation encompasses threshold skills and vocational thresholds. Therefore, medical educators and researchers should be cognisant of the emergent variations in interpretation and terminology associated with the TCF in the context of medical education so they can, for example, include these terms in literature search strategies.

### 4.3 Methodological considerations

This scoping review has demonstrated the vast extent of the current work exploring the TCF in medical education. However, the heterogeneity of the literature presented challenges for synthesis and only broad conclusions can be drawn. There was significant variation in characteristics used to identify possible TCs, which is consistent with findings in the wider literature,^8^, ^64^ and is one of the criticisms of the TCF.^10^ There were also several papers in which the TCF was used to support the topic of discussion but with no evidence to justify its inclusion.^29^, ^31^, ^52^ Although no formal assessment of quality was carried out as part of this scoping review, a lack of experimental rigour was noted in several papers, particularly those focused on the use of the TCF in teaching. Consistent with the wider literature,^64^ there was frequently a lack of evidence to justify the selection of concepts or demonstrate threshold crossing post‐intervention.^30^, ^41^, ^43^, ^51^, ^54^, ^55^ Therefore, the results of these papers should be interpreted with caution.

It is imperative that if ongoing research into TCs is to have a meaningful impact in medical education, researchers must apply high degrees of rigour and transparency in the application of the framework and the design of their studies. This needs to involve clear justification for applying the TCF, an evidence‐based approach to identification or utilisation of specific TCs and replicable methodology.

Further limitations to this review are that papers focusing on health care professionals in general were excluded and some findings applicable to medical education, for example, related to interprofessional working, could have been missed. Additionally, the review focused on education around practicing medicine, and other papers, such as those addressing faculty development, were excluded. However, the role of the doctor goes beyond delivering patient care, and these other professional duties were not captured.

### 4.4 Implications for medical education and research

This review demonstrates how the TCF can provide a lens for examining important transformations learners undergo in their developmental journey at medical school and during clinical practice. Several broad recommendations for medical educators have been identified based on the review findings and are summarised in Table 4. However, further research is needed to develop a consistent approach for describing and applying the TCF to the broad range of learning that occurs during the developmental journey of medical students and doctors. This is especially pertinent given the evolving nature of the evidence base and the fact that new TCs will continue to emerge with advancing medicine.^54^ In addition, how the TCF can be used in teaching and how threshold crossing can be measured are significant gaps in the current evidence and require further research.

**TABLE 4:** Recommendations for medical educators based on review findings

Review findings	Recommendations
TCs in medical education often represent ontological transformations that underpin ways of thinking and practising within medicine.	Identification of these TCs to help learners develop the necessary cognitive skills and attitudes to practice effectively. Identification of learning experiences which help students cross these thresholds and incorporation of these into education and training programmes. Incorporation of TCs into curricula design to help prepare students for real‐world practice.
Recognising and managing uncertainty is a prominent TC for trainees at all stages and is a principal component of other TCs, for example, clinical decision making.	Introduction of learning experiences to help students and trainees develop approaches to uncertainty, for example, shared reflection or mentoring.
TCs related to ways of practising involve learners embodying concepts they may have previously learnt about, such as patient‐centredness.	Introduction of learning experiences that enable students to enact their learning and cross further thresholds, for example, simulation or role‐modelling.
There is individual variability in student experience of TCs and the learning associated with TC crossing is unique to individuals.	Utilisation of the TCF as a lens for identifying students who are struggling to grasp concepts related to ways of thinking and practising.
TCs are recursive across the medical education continuum, particularly occurring at points of transition.	Explaining this to learners to help them prepare for and approach challenging learning. Identification of points where learners may struggle and require additional support. Utilisation of the TCF as a lens for improving continuity across the undergraduate, postgraduate and continuing medical education curricula.
Trainees may progress in training without having grasped important TCs.	Incorporation of TCs into assessment and professional training outcomes.

## 5 CONCLUSION

The TCF has garnered increasing attention in the medical education community in recent years. Much of the focus has been on identifying TCs which underlie the development of disciplinary ways of thinking and practising at various points in training. There was an increasing complexity to the understanding and embodiment of uncertainty, patient care, clinical reasoning and professional identity TCs across the medical education continuum, representing the conceptual changes of knowing, thinking and practising. TCs recurred with changes in clinical environment and responsibilities, endorsing a holistic approach to the curriculum of lifelong learning.

Further research is needed to develop a consistent approach for describing and applying the TCF to the developmental learning journey of medical students and doctors, particularly where new TCs emerge as medicine advances. In addition, how the TCF can be used in teaching and how threshold crossing can be measured are significant gaps in the current evidence and require further research.

## AUTHOR CONTRIBUTIONS

Dr Helen Jones made substantial contributions to the design of the review including the aims, review questions and methods. HJ developed the search protocol and undertook the searches. HJ independently screened the abstract and full texts and met with LH to discuss and resolve any disagreements. HJ charted and analysed the data. HJ and LH discussed the synthesis of the data. HJ drafted the article. HJ gives final approval of the version to be published and agrees to be accountable for all aspects of the work. Dr Lucy Hammond made significant contributions to the design of the review including the aims, review questions and methods. LH independently screened the abstract and full texts and met with HJ to discuss and resolve any disagreements. LH verified the charted data for accuracy. LH and HJ discussed the synthesis of the data. LH critically reviewed and revised the draft article. LH gives final approval of the version to be published and agrees to be accountable for all aspects of the work.

## CONFLICT OF INTEREST

No competing interests to declare.

## ETHICAL APPROVAL

No ethical review is required for this scoping review because it is limited to the review of data that is freely available in the public domain.

## Supporting information


**Appendix S1.** Preferred Reporting Items for Systematic reviews and Meta‐Analyses extension for Scoping Reviews (PRISMA‐ScR) Checklist
**Appendix S2.** Summary of included studies

**Table S1:** Medline (Ovid) search strategy.
