# Supplementary material for: Threshold concepts in medical education: A scoping review
Source: Med Educ. 2022 Jul 24;56(10):983–93. doi: 10.1111/medu.14864 (PMC9543879; doi:10.1111/medu.14864)
Supplement: Supplementary file 2 — Table S1: Medline (Ovid) search strategy. [file MEDU-56-983-s001.docx]

**Table 2: Medline (Ovid) search strategy.**

| Database: Ovid MEDLINE(R) ALL <1946 to June 28, 2021>  Search Strategy:  --------------------------------------------------------------------------------  1 exp *Education, Medical/ (129191)  2 ("medical education" or "undergraduate medical education" or "graduate medical education" or "continuing medical education" or "residency" or "internship" or "clinical teaching" or "clinical education" or "medical teaching").ti,ab,kw. (81385)  3 ((educat* or school* or university or college or curricul*) adj3 medic*).ti,ab,kw. (165409)  4 exp *Students, Medical/ (26620)  5 exp *Faculty, Medical/ (8225)  6 exp *Physicians/ (109626)  7 ((student* or graduate* or pract* or teach* or educat*) adj3 medic*).ti,ab,kw. (163180)  8 1 or 2 or 3 or 4 or 5 or 6 or 7 (441935)  9 "threshold concept*".mp. or ("threshold*" and ("transformati*" or "liminal*" or "troublesome" or "irreversible" or "integrative" or "bounded" or "discursive" or "reconstitutive")).ti,ab,kw. (4375)  10 8 and 9 (56)  11 limit 10 to (english language and yr="2003 -Current") (47)  *************************** |
| --- |
