# Supplementary material for: Threshold concepts in medical education: A scoping review
Source: Med Educ. 2022 Jul 24;56(10):983–93. doi: 10.1111/medu.14864 (PMC9543879; doi:10.1111/medu.14864)
Supplement: Supplementary file 3 [file MEDU-56-983-s002.docx]

### Appendix S1: Preferred Reporting Items for Systematic reviews and Meta-Analyses extension for Scoping Reviews (PRISMA-ScR) Checklist

| SECTION | ITEM | PRISMA-ScR CHECKLIST ITEM | REPORTED ON PAGE # |
| --- | --- | --- | --- |
| TITLE |
| Title | 1 | Identify the report as a scoping review. | 1 |
| ABSTRACT |
| Structured summary | 2 | Provide a structured summary that includes (as applicable): background, objectives, eligibility criteria, sources of evidence, charting methods, results, and conclusions that relate to the review questions and objectives. | 1 |
| INTRODUCTION |
| Rationale | 3 | Describe the rationale for the review in the context of what is already known. Explain why the review questions/objectives lend themselves to a scoping review approach. | 1-2 |
| Objectives | 4 | Provide an explicit statement of the questions and objectives being addressed with reference to their key elements (e.g., population or participants, concepts, and context) or other relevant key elements used to conceptualize the review questions and/or objectives. | 2-3 |
| METHODS |
| Protocol and registration | 5 | Indicate whether a review protocol exists; state if and where it can be accessed (e.g., a Web address); and if available, provide registration information, including the registration number. | 2 |
| Eligibility criteria | 6 | Specify characteristics of the sources of evidence used as eligibility criteria (e.g., years considered, language, and publication status), and provide a rationale. | 3 |
| Information sources | 7 | Describe all information sources in the search (e.g., databases with dates of coverage and contact with authors to identify additional sources), as well as the date the most recent search was executed. | 2 |
| Search | 8 | Present the full electronic search strategy for at least 1 database, including any limits used, such that it could be repeated. | Table S1 |
| Selection of sources of evidence | 9 | State the process for selecting sources of evidence (i.e., screening and eligibility) included in the scoping review. | 2-3 |
| Data charting process | 10 | Describe the methods of charting data from the included sources of evidence (e.g., calibrated forms or forms that have been tested by the team before their use, and whether data charting was done independently or in duplicate) and any processes for obtaining and confirming data from investigators. | 3 |
| Data items | 11 | List and define all variables for which data were sought and any assumptions and simplifications made. | 3 |
| Critical appraisal of individual sources of evidence | 12 | If done, provide a rationale for conducting a critical appraisal of included sources of evidence; describe the methods used and how this information was used in any data synthesis (if appropriate). | N/A |
| Synthesis of results | 13 | Describe the methods of handling and summarizing the data that were charted. | 3 |
| RESULTS |
| Selection of sources of evidence | 14 | Give numbers of sources of evidence screened, assessed for eligibility, and included in the review, with reasons for exclusions at each stage, ideally using a flow diagram. | 4 |
| Characteristics of sources of evidence | 15 | For each source of evidence, present characteristics for which data were charted and provide the citations. | 3-4 & Appendix S2 |
| Critical appraisal within sources of evidence | 16 | If done, present data on critical appraisal of included sources of evidence (see item 12). | N/A |
| Results of individual sources of evidence | 17 | For each included source of evidence, present the relevant data that were charted that relate to the review questions and objectives. | Appendix S2 |
| Synthesis of results | 18 | Summarize and/or present the charting results as they relate to the review questions and objectives. | 3-6 |
| DISCUSSION |
| Summary of evidence | 19 | Summarize the main results (including an overview of concepts, themes, and types of evidence available), link to the review questions and objectives, and consider the relevance to key groups. | 6-8 |
| Limitations | 20 | Discuss the limitations of the scoping review process. | 7-8 |
| Conclusions | 21 | Provide a general interpretation of the results with respect to the review questions and objectives, as well as potential implications and/or next steps. | 8-9 |
| FUNDING |
| Funding | 22 | Describe sources of funding for the included sources of evidence, as well as sources of funding for the scoping review. Describe the role of the funders of the scoping review. | N/A |

### Appendix S2: Summary of included studies 

|  | Arnold et al. (2020)52 | Barron et al. (2020)36 | Barry & Littlewood (2017)46 | Bhat et al. (2018)39 |
| --- | --- | --- | --- | --- |
| Aims and purpose | "...compared the corpus of knowledge and phronesis related to CMEs [context/meaning effects] between Australian graduate medical students and rheumatologists to gain a sophisticated understanding of this relationship to inform curriculum development."(p1) | "To describe the reflections of students who completed the Bushmaster field exercise and to understand threshold concepts they may have encountered."(p2) | "The purpose of the following discussion is to consider the promise of the threshold concepts framework in health care education generally and in anesthesiology specifically."(p1386) | "The purpose of this study therefore was to identify the types of TCs that junior trainees (senior medical students and junior residents) may encounter during their internal medicine clinical rotation and explore their relationship to professional identity formation and clinical task performance."(p621-622) |
| Country | Australia | United States | United States | Canada |
| Medical speciality | Rheumatology | Military medicine | Anaesthetics | Internal medicine |
| Research approach | Online questionnaire. | Qualitative thematic analysis of reflective essays with TCF lens. | Conceptual analysis. | Constructivist grounded theory approach: re-analysis of previously collected data from field observations and field interviews with junior trainees using TCs as sensitising concept; interviews with attending physicians. |
| Participants/ population | Medical students; speciality clinicians. | Medical students. | N/A | Medical students and junior residents; attending physicians. |
| Nature of inquiry | Explains (outlines how understanding of CMEs is important in clinical practice) and Analysis (uses questionnaire results to discuss varying understanding of aspects of CMEs between medical students and clinicians). | Identify (identifies original TC). Discuss (describes the TC in relation to TC characteristics: transformative, integrative, troublesome, and liminality). Explains and Analysis (uses quotes from data to explain who how TC is important in practice). | Identify (names one TC based on own experience and makes suggestions of other potential candidates). Creates (brings TCF into discussion with new body of ideas; suggests ways of identifying TCs; suggests method of teaching TCs). | Identify (identifies 9 original TCs). Discuss (discusses the identified TCs in relation to transformational and troublesome characteristics). Explains and Analysis (uses collected data to explain how identified TCs relate to clinical practice and competence). Appraise (revisits data collected from prior study and re-evaluates through lens of TCF). |
| Paper findings | "Grasping that CMEs occur is a threshold concept in the understanding of the variance in patient responses to therapy. Students and physicians need to be aware when they and the medical encounter may generate CMEs. In this study, a number of these aspects are variably appreciated."(p10) | “In this study, the overarching threshold concept was the transition from being a medical student to a military physician.”(p3) | Named ventilation-perfusion (V/Q) mismatch as a TC amongst medical students based on own experience and discussed teaching approach. Suggested other potential candidates for TCs in anaesthetics which warrants further investigation: interprofessional practice; cognitive load and use of cognitive aids; illness scripts. Discussed the relationship between liminality and imposter phenomenon (IP). Discussed a two-step approach developed by one of the authors for finding possible threshold concepts. Discussed role of simulation in teaching TCs: "Simulation experiences may thus provide the best modality available for addressing threshold concepts".(p1389). Advocated examination of TCs in Entrustable Professional Activities. | Identified 9 TCs, grouped into 3 categories: 1. "Developing as a Professional…refers to TCs that are important for trainees to cross in order to more effectively function as clinical learners". Incorporated: "Active Learner"; "Burden of Responsibility"; and "Plurality of Role" (TC).(p623) 2. "Providing Patient Care… refers to TCs that are important for trainees' interactions with and management of their patients. Incorporated: "Uncertainty in Medicine"; "Purposeful Action"; "Contextual Care"; and "Patient Centredness" (TC).(p623) 3. "Working Collectively… refers to TCs that are important for trainees to cross in order to work effectively within the context of team-based care". Incorporated: "Collective Competence"; and "Documentation as Essential Practice"(p625) There was no apparent influence of training level on TCs crossed, suggesting "trainees can progress in their training but still be struggling with important developmental concepts."(p628) |
| Paper conclusions/ recommendations | “Curricular emphasis is needed to permit an honest assessment of the components that influence when, how and why patient outcomes arise, and how one's agency might have neutral or negative effects but could be inclined towards positive and away from negative patient outcomes."(p10) | “…These are certainly momentous rites of passage, but for the medical students at the USUHS, it is Bushmaster that comes to mind. This is a threshold experience whereby many students attain their new identity as a military physician…”(p4)  Intend to use findings to inform changes to their debrief sessions, student communications (discussing concepts students are likely to experience), and essay prompts. | "Whether adopted incrementally or in total, we suggest that the model of threshold concepts is invaluable in understanding the career development of clinicians... It is now time to quantitatively investigate the effectiveness of the threshold concepts framework in improving learner outcomes. This, in the end, will define the roles of the threshold concepts framework in everyday educational practice and life-long learning".(p1392) | "The identified TCs can therefore support educators to (i) proactively share typical challenges with trainees and help them to work through them and (ii) identify particular trainee behaviours that are less effective and offer insights into potentially troublesome concepts that may be underpinning these. Similarly, TCs may also serve to guide and support assessment practices. More specifically, we could envision their use in the development of entrustable professional activities (EPAs)"(p628) |
| Our findings/ appraisal | No empirical evidence provided to support statement that CMEs meet the criteria for TCs. Role of TCF in authors’ arguments unclear and not supported by evidence. | Analysed themes in relation to transformative, integrative, and troublesome characteristics and provided evidence from data to support this. Discussed liminal space that students occupy but did not provide any specific evidence to support this. TC related to identity formation. Evidence of boundedness. | Identified an original TC based on observation but did not provide any empirical evidence or discuss it in relation to TC characteristics. Novel insight into relationship between TCF and IP. Association is theoretical and so would warrant further research. | Iterative and robust methods used to identify TCs, including different stakeholders. Described TCs in relation to transformational and troublesome characteristics and provided evidence from data set to support this. Explored influence of TCs on clinical practice and related to level of trainee competence – TCs therefore indicated change in practice and development in the profession. Further research required to identify how stronger trainees had gained certain TCs. |

|  | Bhat & Goldszmidt (2020)44 | Blackburn & Nestel (2014)21 | Chen & Poole (2018)45 | Chen et al. (2020)55 |
| --- | --- | --- | --- | --- |
| Aims and purpose | "It is with great pleasure that we write this commentary to the paper by Looman et al which focused on intraprofessional collaboration (IntraPC) amongst health care providers (HCPs)... The study also had us wondering if IntraPC met the criteria for a threshold concept (TC) and, if so, what further implications this would have on the structure of clinical practice."(p1088) | "We sought to explore how “threshold concepts” applied to pediatric surgical training with a view to identifying elements that were “troublesome.””(p756) | Not stated. | “…the goal of this study is to determine whether basic medical blended laboratory courses can help students better understand certain threshold concepts and improve their abilities of self-learning, understanding, and problem solving."(p10) |
| Country | Canada | United Kingdom | Canada | China |
| Medical speciality | N/A | Paediatric surgery | N/A | Biomedical science |
| Research approach | Commentary in response to: Looman N, Fluit C, van Wijngaarden M, de Groot E, Dielissen P, van Asselt D, de Graaf J, Scherpbier-de Haan N. Chances for learning intraprofessional collaboration between residents in hospitals. Med Educ. 2020;54(12):1109-1119. | Grounded theory approach. Semi-structure depth interviews. | Commentary on: Bhat, C., Burm, S., Mohan, T., Chahine, S., and Goldszmidt, M. (2018). What trainees grapple with: a study of threshold concepts on the medicine ward. Medical education, 52(6), 620-631. https://doi.org/10.1111/medu.13526. | Teaching intervention, evaluated via student questionnaires, teacher questionnaires, and comparison of student quiz scores with previous cohort (traditional laboratory course). |
| Participants/ population | N/A | Postgraduate speciality trainees. | N/A | Undergraduate medical students. |
| Nature of inquiry | Identify (names a TC). Discuss (discusses the TC in relation to transformation and troublesome characteristics of TCF). | Identify (identifies areas of troublesomeness, some of which could be TCs). Explains (explores how troublesomeness is important in becoming a paediatric surgeon). | Creates (develops new insight into TCs; and suggests method of teaching of TC). | Creates (uses TCF in teaching). |
| Paper findings | “…we would argue that IntraPC meets the criteria for a TC and that it is a TC that HCPs must cross if we want to improve IntraPC and ultimately patient care.”(p1089). | “Thematic analysis of the transcripts revealed troublesomeness within the areas of: knowledge, clinical judgment, technical skills, transitions between different roles (including validation as a pediatric surgeon), relationships with trainers, and the effect of negative experiences.”(p757) | “…some of the categories in the present study seem more akin to skills or skill sets than to concepts… Some of the types of troublesomeness identified by Bhat et al. … seem to be more closely associated with the everyday angst of becoming a professional than with the realisation that one’s long-held view of something is simply wrong.”(p585) Suggested a method for helping trainees address “uncertainty in medicine” based on their own practice. | "Approximately 71.1% of the students agreed that blended courses improved their understanding of threshold concepts and the underlying theories and 53.76% of the students agreed that their analytic abilities and critical thinking skills were developed via blended laboratory courses online... The majority of the students (63.01%) held the opinion that the blended laboratory courses were better at fulfilling learning objectives, and the modified method helped most of the students (67.63%) better understand the threshold concepts and the underlying theory... The students also commented that traditional lectures might require certain theoretical knowledge that was difficult to understand..."(p11) |
| Paper conclusions/ recommendations | "Although Looman et al have done important work investigating factors in the education of IntraPC, this may not be a problem that can be solved by education alone. Rather, the profession needs to recognise that it is actually the social and material barriers that are shaping practice and the ability (or inability) to engage in IntraPC. We would further argue that there is pressing need to reconceptualise what IntraPC collaboration should be and then enact system changes to ensure that it can be realised in daily practice."(p1089) | “The study shows the importance of troublesomeness in the process of becoming a pediatric surgeon, particularly in the context of negative experiences that trainees have encountered.”(p760). Recommendations included: maximising educational effect of negative experiences, e.g., by discussing mistakes with peers; formal mentoring programme; recognition of liminality by trainers. | N/A | “In conclusion, blended laboratory courses are successful and welcomed by both students and teachers in undergraduate laboratory courses”(p13) |
| Our findings/ appraisal | No empirical evidence provided that IntraPC meets transformative and troublesome TC characteristics and would warrant further investigation. Discussed organisation and structural barriers that make IntraPc troublesome – this is not consistent with TCF. Discussed how other TCs, e.g., uncertainty in medicine, could interact with IntraPc – example of TCs as a network. | Used the lens of “troublesomeness” but this was not extended to the identification of any specific TCs. Aspects of ways of thinking and practicing, including uncertainty, clinical reasoning, and professional identity. Practical skills identified as troublesome. Some aspects of troublesomeness appeared to arise from practical or organisation barriers – this is not consistent with TCF. Small sample size and author was part of the community – potentially affecting data collection and analysis. | Distinguished between TCs and difficult to master skills that can be gained with time and practice. Difference in understanding of TCF between different authors. No empirical evidence provided to support suggested teaching method – would require further investigation. | Homeostasis given as an example of a TC addressed in teaching, but no evidence or reference to justify it as such. Other TCs addressed were not stated – limits reproducibility and application of findings. Evaluation did not ask about specific TCs and term “concepts” was used in one question instead of “threshold concept” – limits validity. No comparison between quiz scores for specific TCs – cannot draw conclusions from data about impact on learning in relation to the TCF. |

|  | Collett et al. (2017)22 | Delaney et al. (2017)51 | Derias et al. (2021)42 | Evgeniou et al. (2015)23 |
| --- | --- | --- | --- | --- |
| Aims and purpose | "In this paper we discuss a research method that allows the application of threshold concept theory to the exploration of the process of 'non-biomedical science learning in medical education'."(p99) | “In the present study, we address this problem by comparing different approaches for teaching the Hardy-Weinberg law (also known as Hardy-Weinberg equilibrium) in large first year genetics classes in undergraduate medicine and science.”(p674) | “Nearly all research into threshold concepts has been from the perspective of teachers. We argue that the students’ perspectives can also be helpful. In this commentary, we explore this issue and inform the debate by drawing on insights from a pilot study in which medical students articulated their own views on what constituted threshold concepts from recent learning experiences in physiology... The study sought to compile a list of TCs that could be used by teachers of medical physiology in particular."(p917,919) | "This project attempts to identify the factors influencing the development of the characteristic ways of thinking and practicing like a microsurgeon… this project will attempt to provide a better understanding of the challenges in microsurgical training and their impact on learning. It will also attempt to provide an insight on what trainers can do to help trainees cross such thresholds and develop the ways of thinking and practicing characteristic of microsurgeons."(p581) |
| Country | United Kingdom | Australia | United States | United Kingdom and United States |
| Medical speciality | N/A | Biomedical science | Medical physiology | Microsurgery |
| Research approach | Ethnographic study. Qualitative thematic analysis of audio-diaries. | Teaching intervention, evaluated via student survey and short answer question in summative examination. | Commentary on pilot study. | Qualitative phenomenological research approach. In-depth interviews. |
| Participants/ population | Undergraduate medical students. | Undergraduate medical students. | Undergraduate medical students. | Postgraduate speciality trainees; trainers. |
| Nature of inquiry | Identify (identifies likely TCs). Discuss (links some likely TCs to named TC characteristics). Explains (discusses the findings in relation to subject teaching). Creates (develops understanding of TC characteristic; suggests a potential new TC characteristic). | Creates (uses TCF in teaching). | Identify (identifies original TCs). Explains and Analysis (explains with reference to student reflections why understanding some of these TCs is important). Creates (uses TCs in a novel way: uses explanation of TCs to help students identify their own TCs and reflect on their own learning). | Identify (identifies potential TCs). Discuss (discusses potential TCs in relation to characteristics of troublesome, transformative, and irreversible). Explains and Analysis (uses interviews with participants to explore importance of TCs in trainee development). Creates (suggests curriculum based on findings of study). |
| Paper findings | “…under the broad theme of ‘being a doctor’ the possible threshold concepts appeared to be: ‘being a doctor’ (referring to the actual embodied lived experience of doctoring) ‘thinking like a doctor’ and ‘uncertainty in medicine’. Under the theme of ‘ethical issues’, ‘medicine is not black and white’ and ‘there is no simple ethically correct answer’ met 3 or more of the criteria. Other themes… were as follows: under the theme of communication skills ‘empathy’ and ‘two way discussions’ were possible threshold concepts. Under the theme of ‘holistic care’, potential threshold concepts included ‘being a doctor is more than just treating symptoms’ and ‘the importance of treating the whole patient’. Finally, within the theme of ‘the patient experience’, ‘patient expectations of doctors’ stood out as a possible threshold concept.”(p109-110) | “Approximately one third of both the Science and Medicine students found the concept somewhat difficult or very difficult… This supports our conclusion from previous teaching experience that the Hardy- Weinberg law is a difficult or ‘troublesome’ threshold concept for many students, and confirms our assumption that it is an appropriate concept for investigating the teaching of difficult concepts in large classes”(p677) “Approximately half of the students in each class enjoyed or really enjoyed the live simulation… and felt that it improved or greatly improved their understanding of the Hardy-Weinberg equilibrium… While many students enjoyed the live simulation and felt that it contributed to their learning, a majority of students in both Science and Medicine reported a strong preference for a ‘face-to-face’ tutorial over a lecture-based simulation. Nearly two thirds of Science and Medicine students felt that the tutorial was more useful than the simulation in improving their understanding of Hardy-Weinberg equilibrium.”(p677) | "Based on the criteria for TCs, a number of potential candidate TCs emerged. Some candidates met all the criteria for TCs. These included secondary messenger systems, pressure gradients, preload/afterload, and cell membrane potential. (TC) Other candidate concepts met some, but not all, criteria. These were the renin-angiotensin-aldosterone system, acid-base homeostasis, and thermoregulation."(p919) | “Trainees and trainers agreed that cognitive skills in microsurgery are much more difficult to develop than practical skills. Preoperative decision making on what kind of flap to use for each patient and how to design a flap, intraoperative decision making, including adjustment of their initial operative plan based on their intraoperative findings, and postoperative decision making, such as in cases when a complication arises, presented as a big challenge for trainees.”(p585) |
| Paper conclusions/ recommendations | “Our study appears to show for example that clinician facilitated small group work can lead to profound shifts in the way that students think about medicine, particularly with respect to the act of being a doctor. We wonder whether a different set of facilitators (patients for example) might help students to explore threshold concepts related to being a patient and provide students with a deeper understanding of the patient experience.”(p111) | “The results of this study provide valuable information on the teaching of threshold concepts in undergraduate Medicine and Science. Students in both courses are divided in their preference for small group tutorials and lecture-based simulations, and so a course that omits one of these teaching approaches (or attempts substitution with a poorly integrated computer simulation) is unlikely to produce good learning outcomes.”(p683) | “Threshold concepts offer a way to improve medical education by drawing attention to key ideas that must be learned but are troublesome for many students. Medical students themselves can provide insights into threshold concepts in biomedical sciences that have the potential to inform curriculum design and pedagogy. These insights can complement work to articulate threshold concepts done by content experts. Informing students about threshold concepts has the potential to help students understand and monitor their own educational experience.”(p920) | “Based on the findings of this study, our proposed microsurgical fellowship training curriculum starts with the development of basic microsurgical skills in the laboratory… the next step in the training curriculum should be the development of practical skills in the clinical setting… Although cognitive skills should be developed in parallel and in conjunction with practical microsurgical skills, they continue to be developed even after trainees have achieved a satisfactory level of technical competence and form the final part of the suggested microsurgical training curriculum”(p586) |
| Our findings/ appraisal | Identified TCs as most likely candidates if they fulfilled 3 or more TC characteristics. The characteristics that the other possible TCs mapped to were not stated. Novel conceptual insights about TCs: that they may not always be bounded, and that transfer may be an additional TC characteristic that indicates mastery. Challenges in identifying irreversibility. Identified audio-diaries as an effective method of research into student perspectives. Appears to arise from same research as Neve et al. (2017) – overlap in findings. | Did not provide evidence that the Hardy-Weinberg law is a TC, beyond it being troublesome – cannot draw conclusions from data about impact of teaching intervention on TCs. Did not compare results pre- and post-intervention. Student preference for teaching format potentially partly driven by familiarity. | Commentary on pilot study - limited information about TCs identified and which TC characteristics they met. Nature of TCs made explicit to students, combined with asking them to apply knowledge before identifying them – appeared to be a successful approach for identifying TCs. Explanation of TCs to students appeared to promote metacognition and appreciation of benefits of deeper learning and could help students take a more active role in their learning. | Comprehensive and robust approach to identifying TCs, including different stakeholders and participants from two different countries. Clear regarding purpose of the study and its limitations – aids interpretation and application of findings. Insightful discussion re. practical skills – appeared to be troublesome and transformative, but difficulties could come from lack of opportunities and skills could be developed relatively quickly by focused training and practice. Therefore, authors did not feel they represented TCs. |

|  | Gaunt & Loffman (2018)32 | Gupta & Howden (2019)34 | Ho et al. (2021)54 | Kamel-ElSayed & Loftus (2018)43 |
| --- | --- | --- | --- | --- |
| Aims and purpose | Not clearly stated. | "We read the recent article by Hilary Neve on threshold concepts involved in becoming a primary care professional with great interest. It prompted us to reflect on our research findings which resonate with some of the issues discussed in the paper."(p325) | "The aim of this study is to report our experiences in the teaching of threshold concepts as well as the perspective and feedback of students."(p1122) | “In this commentary, we use an example where several theories were used to inform an educational intervention. These theories include constructivism, threshold concepts, narrative medicine, dialogism, and active learning. We also outline how these ideas align with each other.”(p255) |
| Country | United Kingdom | United Kingdom | Taiwan | United States |
| Medical speciality | N/A | General Practice | Surgery | Biomedical science |
| Research approach | Conceptual analysis. | Comment on: Neve H. Learning to become a primary care professional: insights from threshold concept theory. Educ Primary Care. 2019 Jan 2;30(1):5-8. Re-appraisal of findings from their own previous study in light of the paper, through the lens of TCs. | Teaching intervention, evaluated via student questionnaires and essay assignment. | Commentary. |
| Participants/ population | N/A | Undergraduate medical students. | Undergraduate medical students. | Not stated but presumed to be medical students – context of paper is medical education and teaching delivered to students. |
| Nature of inquiry | Identify (suggests TC based on own experience). Explain (explains how TC helped students learning). Creates (suggests enhancement of curriculum by teaching students about TCs and encouraging identification). | Identify (identifies TC). Explains and Analysis (discusses student perspective on experience which highlights importance of TC to clinical practice). Creates (suggests ways of teaching TCs). Appraise (re-evaluates previous study findings through the TC lens). | Creates (uses TCF in teaching practice). | Identify (names a potential TC). Describe (describes the TC in relation to transformative and integrative characteristics). Creates (used TCF to inform teaching practice; discussed TCF in relation to other theories). |
| Paper findings | “Examples from the authors’ own teaching practices in medical education include the fact that an X-ray image is a 2D representation of a 3D structure with overlapping tissues of varying densities producing ‘composite shadows’. Only once students recognise this and the range of permutations in appearance of a ‘normal’ body part, can they start to identify the ‘abnormal’.”(p789) | "Viewing our students' experiences and their accounts through the lens of threshold concept framework, 'learning the skill to deal with uncertainty' appears transformative, troublesome initially, integrative and quite likely to be irreversible."(p325) | “In Q1 (filled after watching the online video but before face-to-face discussion), 18.9% (24/127) students reported that all concepts were clear to them and 8.7% (11/127) reported that all concepts were insufficiently clear and required further explanation… The proportion of students who expressed a ‘total change’ or ‘large change’ in their conceptual understanding ranged from 22.0% (in TC3) to 34.6% (in TC1). Therefore, at least 22.0%-34.6% of students benefitted from the online video before the face-to-face class… After the face-to-face class, the proportion of students who expressed that the concepts became clearer than before ranged from 43.5% (TC6) to 75.3% (TC1).”(p1123-1124) | “It could be argued that in the biomedical sciences the concept of homeostasis is an example of a threshold concept that underpins nearly all of human biology… In the example we give below, the threshold concept was homeostasis as it applies to the activity of the thyroid gland.”(p256) “Following the session, a preliminary study was conducted to evaluate the learners’ perceptions by using both questionnaire and focus groups. Feedback was very positive…”(p257) |
| Paper conclusions/ recommendations | “…we suggest that teaching medical students about threshold concepts could help to develop the adept, flexible and fearless approach to lifelong learning that is and has always been required of medical professionals… Medical students could be asked to identify and justify threshold concepts in their curricula and to produce accounts of how they crossed those thresholds as resources to support self-efficacy and to offer explanatory resources for future students from those who have more recently crossed those thresholds.”(p790) | “Longitudinal placement in a nurturing team in LIC allows recursiveness which aids in integration and successful management of liminal spaces… We wholeheartedly agree with the author that practising effectively in primary care involves significant transformative shifts. LIC exposure, we would like to add, provides a stable and safe environment for medical undergraduates to negotiate several thresholds.”(p325-326) | “Threshold concepts were understood differently depending on the concept and individual. We consistently observed that some threshold concepts were grasped better than others. Students can auto-reflect on the defining features of threshold concepts. Although high degrees of satisfaction were reported, students were aware of their deficiencies in knowledge. Few students were sufficiently confident to manage patients in the future.”(p1125) “…threshold-concept-based learning may have applications in course designs based on nonformal learning. In our setting and course design, students could learn at their own pace by either reading or watching the online video focusing on the extracted threshold concepts. This ‘nonformal learning’ strategy can ‘prime’ some students for concept consolidation and may induce ‘phase transition’ experiences in some other brilliant students.”(p1125) | “The main point we wish to make is that by aligning the different theoretical levels and combining insights from several educational theories, we were able to come up with a teaching/learning design that had real theoretical depth and allowed us to analyze what needed to happen, and what did happen, and how we can improve it in the future.”(p258) |
| Our findings/ appraisal | No empirical evidence provided to support suggested TC – would require further investigation. Suggestions about benefits of teaching students explicitly about TCs – would warrant further research. | Provided evidence from primary data to support identification of TC but did not describe how it corresponded to TC characteristics. TC related to uncertainty. Clerkships as a space to address TCs – would require further research. | TCs to guide design of teaching intervention were identified by clinical teacher – no empirical evidence provided in justification. TCs were phrased as statements of fact or recommended clinical practice and so it could be argued do not meet the definition of “concept” – limits conclusions that can be drawn from paper related to teaching interventions for TCs. Some student comments provided lacked sufficient detail to support authors’ interpretations. Idea of “troublesomeness” missing throughout study results and discussion. Authors appeared to suggest “troublesomeness” was synonymous with “bounded” – adds confusion. Novel insight into evolving nature of medical practice results in evolving TCs. | New conceptual insight by using multiple learning theories, including the TCF, in planning and delivering a teaching session. Argued that homeostasis was a TC but did not reference this or provide any empirical evidence. Limited discussion of outcomes of intervention on student learning. Outcomes of intervention cannot solely be attributed to the TCF due to combined approach. |

|  | Khatri et al. (2020)24 | Khurshid et al. (2020)53 | Manson & Jones (2020)31 | Meyer et al. (2020)37 |
| --- | --- | --- | --- | --- |
| Aims and purpose | "The purpose of this study is to identify and understand threshold concepts that are central to the mastery of psychiatry at the undergraduate level. We also discuss educational activities that could support students through the transformations they undergo."(p179) | "This review attempts to fill the gap in literature relating to students' way of thinking and practising prescription writing. It throws light on the troublesome knowledge and various other determinants of prescription writing that can influence epistemological transitions and ontological transformations of a novice learner.”(p2) | "This exploratory study aimed to determine how senior educators' beliefs might influence psychological preparedness training."(p1) | "To describe and understand the themes and perspectives of the works submitted by health care students".(p2) |
| Country | United Kingdom | Australia, Pakistan, Oman | United Kingdom | United States |
| Medical speciality | Psychiatry | N/A | N/A | Military medicine |
| Research approach | Curriculum enquiry rooted in grounded theory: semi-structured interviews with psychiatry educators and questionnaire sent to medical students. | Narrative review. | Mixed research approach including conceptual analysis and empirical aspect (elite interviews). | Qualitative thematic analysis of reflective essays, stories, poems, and artworks published in a peer-reviewed literary magazine. |
| Participants/ population | Undergraduate medical students; medical educators. | Undergraduate medical students. | Medical educators (in context of undergraduate medical student education). | Medical students. |
| Nature of inquiry | Identify (identifies 3 original TCs). Discuss (describes TCs in relation to 5 TC characteristics: transformative, troublesome, integrative, irreversible, and bounded). Explains and Analysis (explains how identified TCs address educational issue with relation to evidence collected). Creates (suggests teaching methods to address identified TCs). | Identify (identifies possible TC or topic with several underlying TCs). Discuss (describes topic in relation to 8 characteristics of TCs: troublesome, transformative, integrative, irreversible, bounded, liminality, discursive, and reconstitutive). Explains and Analysis (explains how topic addresses important issue in education and clinical practice, with reference to evidence from literature). | Identify (names a potential TC) | Identify (identifies 5 TCs). Explains (explains how identified TCs are important for medical education). |
| Paper findings | “We identified the following threshold concepts in this study: Therapeutic Risk-Taking, Concept of Diagnosis within psychiatry, and the Biopsychosocial Model… When students grasp these threshold concepts, they will understand the process of making clinical decisions, balancing risks, and adopting a holistic, patient-centered approach – all of which are important attributes of a competent, safe and caring future doctor.”(p183) | “Prescription writing as a competency transforms a medical student into a prescribing physician through the synthesis of both required knowledge and regulatory elements. The transformation depends on how a medical student integrates the basic principles/knowledge of the discipline and individual characteristic of the drugs for the benefit of the patients considering the regulatory guidelines associated with a balanced prescription.”(p6) “There remains the possibility of a few threshold concepts underlying the facets of prescription writing. Identifying these can help students transform their thinking and open a new way to understanding. Therefore, a balanced approach to prescribing not only entails a critical and proactive way of thinking and practising but also requires mastery of formerly-unidentified threshold concepts.”(p7) | “This study has exposed the concept of resilience as ‘troublesome knowledge’… This suggests that resilience is a ‘threshold concept’ needing ‘a transformed way of understanding...or viewing [the concept], without which the learner cannot progress’. By identifying resilience as a threshold concept in the context of medical education it is hoped that this study will stimulate research into better understanding the obstacles involved in the psychological development of doctors, enabling these to be addressed and overcome.”(p22-23) | “Through our analysis, we were able to get a snapshot of the students’ experience as they faced different threshold concepts important to their professional identity formation. Threshold concepts of "I am a healer," "I can deal with ambiguity," "As a military medical officer, I serve two masters," "Patient is the focus," and "Physicians have a unique and complex relationship with death" were consistently noted throughout our review.”(p8) |
| Paper conclusions/ recommendations | “To support students’ mastering of threshold concepts, we developed pedagogical recommendations to incorporate in a medical school curriculum: student Balint groups exploring therapeutic risk, student Balint groups exploring the role of a psychiatrist, exposure to psychological therapies/ psychotherapy skills, explicit diagnostic reasoning.”(p185) | “Bringing elements of threshold concepts framework in revamping the prescribing process in medical education can ensure a more rational and safe approach to prescription writing. The comparison between different facets of prescribing and threshold concepts can offer the threshold concepts framework as a pedagogical utility in addressing challenges associated with teaching, learning, and assessment of prescription writing at the undergraduate medical educational level. Furthermore, it also offers food for thought to identify the potential threshold concepts inherent in the process of prescription writing.”(p8) | “As resilience is a threshold concept, identifying and addressing troublesome knowledge in students and educators could improve psychological preparedness provision.”(p23) | “A medical school literary magazine, far from being a frivolous extracurricular activity, is, in fact, a needed part of the underlying curriculum of the school, because it allows students to digest and grapple with what it means to a physician.”(p8) “Using the lens of thresholds concepts allowed us to see that some of the themes met the criteria for threshold concepts, in that they were transformative, integrative and troublesome.”(p8) |
| Our findings/ appraisal | Robust study incorporating both students and educators. Limitations acknowledged.  Provided evidence from data to support identification and clearly described all TCs in relation to 5 characteristics.  Suggested approaches to address TCs – would require further research. Recognised individual variation between students’ experiences of TCs. Themes of uncertainty and holistic care. TCs related to ways of thinking and practising. | Looked at 8 characteristics of TCs and used evidence from literature to provide examples of where they applied to prescription writing – different approach from studies using primary data. Thorough discussion of importance of topic for clinical practice and patient safety. Did not state conclusively that prescription writing is a TC and suggested several yet unidentified TCs may be involved – opens up conversation and invites further research. Relates to ways of thinking and practising. | Troublesome knowledge and TCF introduced in final paragraph of discussion. Resilience not examined in relation to any other TC characteristic (including transformative) and no empirical evidence to support identification – further research would be required to establish if it is a TC. Discussion of the implications of psychological preparedness as a TC on medical education is limited. Recommendations lack specific suggestions of how to identify and address troublesome knowledge and how that can be used to improve psychological preparedness provision. | Used a systematic approach to identifying themes in data. Large sample size. Limitations discussed. Did not explicitly describe identified TCs in relation to characteristics and did not provide further evidence from data to support TCs. TCs related to professional identify formation, putting the patient-first, and uncertainty. Challenges in identifying irreversibility. |

|  | Miles et al. (2020)35 | Mitchell et al. (2012)41 | Moeller & Fawns (2018)40 | Neve et al. (2017)25 |
| --- | --- | --- | --- | --- |
| Aims and purpose | N/A | "We designed a structured, fresh cadaver dissection course that focused on providing surgical trainees an opportunity to dissect vascular structures rarely seen in the current era of vascular surgery training... We hypothesized that participation in the course would improve residents' understanding of complex anatomic relationships, improve their confidence in performing these infrequent exposures, and enhance vascular procedural understanding."(p1188) | "Our goal was to identify and explore potential threshold concepts and other troublesome knowledge in EEG learning. Further, we aimed to understand teachers' perspectives on barriers to learning and ways in which students could be supported as they engage with TCs and troublesome knowledge in EEG. Although our primary aims were to explore TCs and troublesome knowledge in EEG learning, we hoped that our findings could provide insights into teaching and learning of other conceptually difficult topics in the health sciences."(p388) | "The aims of this study were to (1) explore threshold concepts associated with professionalism and (2) identify factors that might enable students to 'get' such concepts. A further objective was to contribute to the emerging literature on threshold concepts."(p94) |
| Country | United Kingdom | United States | United States and Canada | United Kingdom |
| Medical speciality | Psychiatry | Vascular surgery | Neurology | N/A |
| Research approach | Letter to editor in response to Khatri R, Knight J, Wilkinson I. 2020. Threshold concepts: a portal into new ways of thinking and practising in psychiatry. Medical Teacher, 42(2):178-186. | “Prospective observational cohort analysis with preintervention and postintervention measures”(p1188). Oral board examination style testing of knowledge, self-reported confidence, and satisfaction evaluation form. | Semi-structured elite interviews. | Naturalistic method of enquiry. Phase 1: qualitative thematic analysis of audio-diary recordings from students and facilitators with reference to TCF. Phase 2: discussion groups with students and tutors. Phase 3: thematic analysis of Phase 1 and 2 data and identification of TCs. |
| Participants/ population | Undergraduate medical students. | Postgraduate residents. | Medical educators (target population of discussion not stated). | Undergraduate medical students; medical educators. |
| Nature of inquiry | Creates (suggests way of teaching TCs) | Creates (uses TCF in teaching). | Identify (identifies one original TC). Discuss (discusses TC in relation to 5 TC characteristics: transformative, probably irreversible, integrative, bounded, and troublesome). Explains and Analysis (explains how TC addresses educational issue, using data collected from interviews). Creates (discusses findings in context of threshold capabilities). | Identify (identifies 7 possible TCs). Discuss (describes how the TCs are related to some of the characteristics, varying depending on the TC). Explains and Analysis (uses data from study to discuss how TCs address an educational issue). Creates (identifies positive teaching and learning experiences for TCs; develops understanding of TC characteristic). |
| Paper findings | “Therapeutic risk-taking is a threshold concept we relate with greatly… At the university we attend, efforts have been made to ensure students master this threshold concept by undertaking group case presentations. Students are encouraged to integrate previous ethics and law teaching into clinical practice while considering different therapeutic viewpoints, and their associated risks, in patient management. This is an alternative method to help students evaluate therapeutic risk and perhaps a more appropriate method for those who find it difficult to discuss openly in Balint groups.”(p840) | “Open vascular surgical operations involve many complex anatomic relationships that are troublesome to understand and are encountered relatively infrequently in traditional clinical training. The course that we developed is an effective method to teach these relationships to the surgical novice, thus lowering the threshold for learning to occur. This fresh cadaver course provides learners a different way of thinking about vascular anatomy than that learned from a surgical atlas or an anatomy textbook. It provides learners an opportunity to view perhaps “troublesome” concepts in a new way that may be “transformative, irreversible, and integrative.”(p1192-1193) | “Polarity emerged as a threshold concept that was identified in every expert interview, usually as the first concept that was discussed. Two other themes of troublesome knowledge – pattern interpretation and clinical significance – contained some features of threshold concepts, but we felt that they did not clearly satisfy all criteria.”(p389) | 7 themes were identified from Phase 1 and 2 which represented possible TCs related to professionalism: 1. "There is a professional culture and I am becoming part of it"(p97); 2. "Consider the whole person"(p98); 3. "I don't need to know everything"(p99); 4. "Consider the bigger picture"(p99); 5. " We have to work with uncertainty"(p100); 6. "People have different expectations"(p100); 7. "Emotional intelligence”.(p101) Themes were identified related to factors which appeared to facilitate transformation and exploration of troublesome areas and emotions: "The safety of the small group setting"(p101); "The opportunity for learners to reflect and discuss issues in a group and to consider different perspectives"(p101); "'Understanding the relevance of their learning', for example, by relating to workplace experiences and scenarios"(p102); "Prompts from the facilitator"(p102); "Conceptual models and frameworks were mentioned as integrating and giving shape to difficult, woolly or opaque ideas"(p102); “Time, multiple and different experience”.(p102) |
| Paper conclusions/ recommendations | N/A | “We firmly believe for vascular surgical training to move forward, it must do so in a more scientific and quantitative fashion. To be on the forefront of surgical education, vascular surgery needs to move in a direction of scientific development and evaluation of curriculum, quantitative assessment of vascular resident competence and performance, and quantitative and objective evaluation of the effectiveness of new and traditional vascular surgical training methods and pathways. This study provides an example of such an approach.”(p1193) | “We have shown how each of these concepts may relate to each other as a threshold conception of EEG learning: polarity forms the core of EEG understanding, and needs to be understood before learners can understand pattern interpretation or determine clinical significance. Our hope is that these findings have clarified potential barriers to learning EEG, ways in which learners can be supported, and potential areas of emphasis in curriculum design. Learners may oscillate between mimicry and genuine understanding, and the learning process requires an acknowledgment of this, with frequent opportunities for repetition and discussions with experts. An acknowledgement of the variability in experiences that students bring to the learning process (pre-liminal variation) can go a long way to supporting students in overcoming barriers to learning. In addition, these TCs and troublesome knowledge may get to the root of expertise; determining whether a student has truly understood a threshold concept may be a useful foundation for assessment of the very highest levels of knowledge and skills in EEG interpretation.”(p393) | “This research, which aimed to describe and ‘stay true’ to student and facilitator experiences of learning offers new perspectives on students’ learning around professionalism. Seeing the relevance of learning to their future professional roles by reflecting on placement experiences in a safe, reflective, small group setting seemed to facilitate the crossing of thresholds. The use of audio-diaries, supported by groups, can help identify threshold concepts in these areas and could be used more widely in other contexts. We identified a number of likely threshold concepts, which were relatively un-bounded, yet fundamental to thinking and practising as a doctor.”(p106) |
| Our findings/ appraisal | Adds student perspective to suggestions made in the original paper. | TCF was referenced in the discussion and no evidence was provided that complex anatomical relationships met TC characteristic or were TCs. Practical barriers which contribute to troublesomeness were discussed – this is not consistent with TCF. No evidence provided to support statement that the course allowed participants to view concepts in ways that are transformative, irreversible, and integrative. No suggestions that participants were assessed for or asked about evidence of transformation or integration – cannot draw conclusions from data about impact of teaching in context of TCF. | Robust methods and a range of participants from different institutions. Limitations acknowledged. Provided examples from data to correlate identified TC with 5 TC characteristics. Identified TC important to understanding other troublesome areas, which were skills or capabilities. Findings linked to Threshold Capability framework. | Robust methods exploring both educator and student perspectives. Limitations addressed. Small sample size but large number of audio-recordings allowing in-depth exploration. Stated that identified TCs met 5 TC characteristics (transformative, integrative, troublesome, liminal, and irreversible) but did not provide evidence of all characteristics for each TC. Sub-concepts underlying TCs could have been steps or TCs themselves – insight into nature of TCs as being inter-related networks. TCs related to ways of thinking and practising. TCs related to uncertainty, holistic view, and professional identify formation. Novel conceptual insights about TCs: that they may not always be bounded. Identified audio-diaries as an effective method of research into student perspectives. Appears to arise from same research as Collett et al. (2017) – overlap in findings |

|  | Neve et al. (2020)28 | O’Callaghan et al. (2020)48 | Pinnock et al. (2019)49 | Randall et al. (2018)38 |
| --- | --- | --- | --- | --- |
| Aims and purpose | "The aim of this study was to work with experienced population health medical educators to identify and explore possible threshold and troublesome concepts in population health."(p293) | "The aim of this study was to explore the experience of doctors undertaking an attachment in palliative care in order to propose a series of specific TCs for doctors learning in this setting."(p423) | “The aim of our study was to investigate whether or not students’ learning and understanding of clinical reasoning could be explained using the threshold skill schema.”(p684) | "We will examine TCs we identified in our pediatric clerks".(p2) |
| Country | United Kingdom | New Zealand | New Zealand | United States |
| Medical speciality | Population health | Palliative care | N/A | Paediatrics |
| Research approach | Transactional curriculum enquiry: workshop with educators to identify potential TCs, followed by questionnaire with quantitively and qualitative elements. | Semi-structured focus group interviews. | Interviews. | Qualitative approach rooted in grounded theory via analysis of reflective essays. |
| Participants/ population | Medical educators (in context of undergraduate medical education). | Postgraduate registrars. | Undergraduate medical students. | Medical students. |
| Nature of inquiry | Identify (identifies possible TCs). Explains (explains how the concepts are important in medical education). | Identify (identifies 5 TCs). Discuss (describes identified TCs in context of some TC characteristics, varying depending on the TC). Explains and Analysis (uses data from study to explain how the TCs addresses an educational issue). | Identify (identifies original TCs). Discuss (discusses TCs in relation to some TC characteristics). Explains and Analysis (draws on findings from data and literature to explain why TCs address an educational issue). Creates (uses lens of threshold skills). | Identify (identifies 7 TCs and one overarching TC). |
| Paper findings | “A total of 29 concepts were identified from participant responses as being possible threshold concepts in population health. As some concepts were closely related or overlapped, the authors… negotiated and agreed a set of 20 concepts on which to base the subsequent questionnaire”(p293) “Attributing causality, inequalities in health and doctors’ responsibility for populations not just individuals were the concepts most participants thought were threshold”(p292) “Uncertainty, evidence-based practice, social justice and the biopsychosocial model have been suggested in the literature as possible threshold concepts and these also map onto our findings.”(p295) | “From this process five TCs were proposed: Emotional Engagement (8 characteristics) Communication Management (8 characteristics) Embodied Shared Care (4 characteristics) Active Inaction (4 characteristics) Uncertainty Embraced (4 characteristics)”(p424) | “We found evidence that clinical reasoning has four features of a threshold skill. We found strong support for “transformation”, “troublesomeness,” “integrative,” and “associated with practice.” We did not find sufficient support to report “semi-irreversibility” as a threshold skill feature of clinical reasoning, however, an alternative feature, “issues with transferability,” was identified.”(p684) | The authors identified 7 TCs: "Being smart isn't enough"; "It's about the patient"; "Life isn't fair"; "Sometimes there isn't a right answer"; "You can't save everyone"; "Learning is lifelong"; "Medicine isn't black and white, but almost grey".(p3-4) "The overarching theme of the threshold concepts is that "There is a disconnect between what I thought medicine was going to be and the reality."  This threshold concept was identified in this and every subsequent data set, and by each medical student co-investigator as the core of what they encountered during their clerkship year."(p4) |
| Paper conclusions/ recommendations | “We believe our findings can be empowering for both teachers and learners. The findings could also inform the development of a population health curriculum framed around threshold concepts and designed to engage learners and support them through the liminal space and over these thresholds.”(p297) | “The registrars in this study identified that their palliative care attachment provided a unique learning opportunity, with skills that were transferrable and valuable to their later practice. Five threshold concepts acquired during their attachment were identified. Further understanding is needed about the attributes of the learning environment required for this learning to occur and whether this can be replicated in non-palliative care settings. These threshold concepts can be used to inform curriculum design and educational support for palliative care attachments. The findings may also have implications for medical education more broadly.”(p426) | “Using threshold skills as a lens for examining clinical reasoning adds an additional framework to the established theories currently available to medical education researchers in their study of clinical reasoning… Similar studies at other institutions are needed to verify whether students’ experiences of learning clinical reasoning share similar threshold features and how these features might be present in medical students at other levels of study. It will also be important to investigate if there are other threshold features associated the development of clinical reasoning. We foresee that this new way of conceptualizing clinical reasoning that may provide productive avenues for pursuing future teaching, learning, and research.”(p688) | "Our work and that of Collett and Neve illustrate the synthetic themes found by Barradell, especially that of working anew with knowledge in the health sciences ("Being smart isn't enough") and induction into the community of practice ("Medicine isn't black and white").  These TCs may be universal to preparation for the responsibility of caring for patients. Alternately, these TCs may vary in medical school settings in societies very different from our own... We will be looking to partner with a medical school with a different culture, to determine which of these TCs are indeed universal inmoving from student to physician-hood"(p5) |
| Our findings/ appraisal | Small sample size but participants from several different medical schools and professional backgrounds resulting in a diversity of perspectives. Limitations acknowledged.  Potential TCs identified discussed with reference to literature, but no exploration of participants reasoning. Only explored transformative and troublesome characteristics and there was a disconnect between concepts felt to be transformative and troublesome – the potential reasons for this were not explored. TCs related to ways of thinking and practising. Discussed challenges in identification due to evolving TCF. | Robust methods. Limitations acknowledged including small sample size. Evidence from data provided to support TCs, but specific characteristics each TC was mapped to were not clearly stated and examples were not provided for every characteristic. Used “recursive” as a characteristic but not “discursive” – variation in application of TCF. TCs may be applicable in other settings – transferability. Environment of palliative care important in helping learners cross TCs. TCs related to ways of thinking and practising, and participants embodied concepts in practice. TCs related to patient-centredness and uncertainty. Participants revisited older learning and re-entered liminal space. | Different approach to other studies as started with a topic of interest and collected data to see if it met certain characteristics. Limitations acknowledged including small sample size from single institution. Identified issues with transferability but not clear if this is a new characteristic. Used perspective of threshold skills instead of TCs – highlights questions of the difference between skills and concepts. Sub-concepts/themes discussed (e.g., dealing with uncertainty, analysing data) – not clear if these are steps or TCs/threshold skills in their own right. | Large sample size. Potential impact of student researchers only considered in positive light. Used evidence from data to support TCs identified. Considered transformative and troublesome essential characteristics but did not state which other characteristics the TCs correlated with. Drew similarities between TCs identified with those found by Collett et al. (2017) – strengthens evidence of findings. TCs related to ways of thinking in the profession. TCs related to ambiguity and patient-centredness. No discussion of why identified TCs are important in educational and clinical practice. |

|  | Santisteban-Espejo et al. (2020)57 | Sharma et al. (2017)30 | Smith et al. (2018)47 | Tredinnick-Rowe (2018)33 |
| --- | --- | --- | --- | --- |
| Aims and purpose | "Our hypothesis is that the use of bibliometrics, as a tool to discover hidden relations between keywords, can overcome traditional difficulties related to TC identification... Our challenge is to identify the bibliometric indexes that are able to show the relationship between the keywords that make them TC, especially in a medical context. In previous scientific literature, several methods were applied, mainly based on qualitative assessment. In this sense, we propose a quantitative, objective, and reproducible approach that can enrich the learning process from a scientific-based perspective."(p1) | "In this article, we illustrate our experience of designing and conducting a totally online course in clinical transplantation... The aim of this article is to assess the performance of educators and how well the ‚'ethos of critical threshold' has been accepted from the perspective of students."(p303,305) | "We aimed to answer the following questions with respect to junior consultant cardiothoracic surgeons: 1. What are threshold concepts for cardiothoracic surgeons, especially in their transition to consultant practice? 2.How may these threshold concepts be negotiated and overcome? 3.How do these threshold concepts influence the acquisition of a cardiothoracic surgical identity? 4.What are the implications of these threshold concepts for the cardiothoracic surgical training curriculum or for the professional development of consultant cardiothoracic surgeons?"(p80) | "This essay sets out to explain how educational semiotics as a discipline can be used to reform medical education and assessment... In this paper, I provide background information about medical education, paradigm shifts, and the concept of suprasubjectivity in relation to modern educational semiotic literature. I conclude by giving examples of what a suprasubjective approach to medical education and assessment might look like. I do this by drawing an equivalence between the notion of threshold concepts and suprasubjectivity, demonstrating the similarities between their positions."(p491) |
| Country | Spain | United Kingdom | Australia | United Kingdom |
| Medical speciality | N/A | Transplant science | Cardiothoracic surgery | N/A |
| Research approach | Theoretical paper. | Programme evaluation. | Constructivist qualitative approach: semi-structured in-depth interviews. | Conceptual analysis. |
| Participants/ population | N/A | Postgraduate medical education. | Junior consultants. | N/A |
| Nature of inquiry | Creates (offers new insight into the identification of TCs). | Creates (uses TCF in curriculum design). | Identify (identifies TCs). Discuss (describes to a certain extent how some of the identified TCs relate to the TC characteristics). Explains and Analysis (uses examples from data to explain how TCs address an educational issue). | Creates (brings the TCF into conversation with a new body of ideas in relation to medical education and assessment). |
| Paper findings | “…advanced bibliometrics methods may be used to achieve the goal of discovering the special charasteristics [sic] of a keyword and to give it the status of TC. The relative importance of the keywords can be discovered by means of the two main methods in bibliometrics, performance analysis and science mapping.”(p2) | “Students’ survey (Kirkpatrick level 1) performed for module 1 of cohort 1 reported students’ satisfaction rate of 93%. Excluding a total of 12 drop-outs from first cohort… as many as 93% of students of first cohort passed module 1 and 100% passed module 2 (learning Kirkpatrick level 2)”(p307) “We promoted highly rated coursework to be published in peer-reviewed journals, also for abstracts based on excellent assignments to be submitted to international meetings… At the time of writing this article, 22 articles were published in peer-review journals and 15 abstracts were presented in international conferences… This may help in promoting the international ranking of our institute that can be interpreted as equivalent to “change in behavior positively affecting the organization” (i.e., at Kirkpatrick level 4).”(p307-308) “All those who passed module 1 (both cohorts) and 2 (1st cohort) registered for their respective next module (return on investment Kirkpatrick level 5).”(p308) | “Consistent with the only previous study of threshold concepts in practising consultant surgeons (Land and Meyer 2011), uncertainty has emerged as the major threshold concept in cardiothoracic surgeons in their transition to consultant practice. Uncertainty has been reflected in each of the eight major challenges described by the surgeons. This study also confirmed the suggestion by the same authors that the speed of operating and the handling of surgical complexity could be troublesome for surgeons. Additionally, we have identified change as a possible threshold concept.”(p88) “In general, the threshold concepts and the associated challenges especially with respect to the actual conduct of cardiothoracic surgery were handled through having the requisite theoretical knowledge, preparing thoroughly for any challenging procedure, and by seeking the advice, assistance or reassurance of experienced surgical colleagues. The accumulation of experience and reflection upon this experience over time resulted in a successful transformation in learning. Some individual variation in how the threshold concepts were negotiated was demonstrated.”(p89) | “Semiotically, what are here described as threshold concepts, are the processes by which a student cognitively begins to access suprasubjective sign relations, that allow a differing perception of self, and one’s wider environment. The student undergoes an ontological shift in relation to their own being, but also potentially in relation to the external world as well…”(p504) |
| Paper conclusions/ recommendations | “In conclusion, we elucidate the application of bibliometrics as a base or a new methodology for the identification of medical TC to better adjust course and curricula design to the inherent complexes and uncertainty conditions of the current medical profession… Thus, medical lecturers could also explore the pedagogical applications of data contained in scientific documents (i.e. keyword analysis) and to implement them at university and research levels. It would be worthy to apply this original approach in the field of advanced therapies and, specifically, in tissue engineering.”(p3) | “Critical thresholds are crucial to understanding a subject and gaining expertise and, therefore, are key to aligning with learning objectives is a properly designed and blueprinted distance learning course. These are the corner stones of critical decision-making in safe and effective clinical practice, the main ethos of this unique totally on-line course in transplantation that delivers clinical education online. We strongly believe that adopting the principle of critical threshold while linking to theoretical knowledge and clinical experience in our pedagogy is the corner stone of success.”(p310) | “Each of the eight challenges could serve as a curriculum target within cardiothoracic surgical education. Workshops covering cardiothoracic surgical decision-making, the various relationships in new work environments, and handling the emotional impact of adverse events, for example, would be welcome. Robust discussion of these issues amongst senior trainees and junior consultants within cardiothoracic surgery and across other specialties would highlight many of the unseen or unspoken issues. Some of the transcripts analysed in this study could form part of the educational materials for these workshops… Once in practice, the junior cardiothoracic surgeons paid tribute to the advice, assistance, and reassurance provided by their more senior colleagues. It is likely that a more formal mentoring programme might further enhance the interaction between junior and senior cardiothoracic surgeons.”(p91-92) | “At the beginning of this paper, the question was asked what attributes and skills are required to make a good doctor. Having established that the quantification of assessment and outcome data can only take us so far in answering this interrogative, one can see why there is a desire for a paradigm shift in medical education, where one can both qualify and quantify the answers to this question. The qualification is best found in approaching medical education and assessment from a reflective, humanistic position, specifically by designing educational practices and assessments that situate medical students’ learning within a suprasubjective context of sign relations. A practical example of this is the use of threshold concepts to determine the critical events that students pass through on their journey to becoming clinical professionals.”(p509) |
| Our findings/ appraisal | New insight into the identification of TCs. Theoretical paper and no evaluation of proposed methodology – would require further research. Lack of detail about what the methodology would look like and how it could be enacted – makes evaluation and application of proposals difficult, particularly for those unfamiliar with bibliometric methods. Focus on studies undertaken in other disciplines and limited analysis of methodologies in medical education research. Translation/language issues made understanding and interpretation of this paper difficult and some of the intended meaning may have been lost. | Challenging paper from which to chart data due to structure and writing style. Evaluation methodology (e.g., in relation to Kirkpatrick levels) and conclusions drawn warrant critical appraisal. Significant lack of citations throughout the paper and references only introduced at the discussion stage. TCs for each module not provided. Learning experiences that were stated were discussed to a certain extent in context of the TCF, but no evidence provided to support the use of these learning strategies. No evaluation of the effectiveness of the learning strategies or course in helping students cross TCs – no evidence to support conclusion that incorporation of TCs is part of the basis for successful outcomes. Terms “threshold concepts”, “critical thresholds”, “crucial thresholds”, “threshold attitudes”, and “threshold skills” used interchangeably throughout. “Threshold” also used in context not consistent with TCF, e.g., in relation to practical barriers – undermines confidence in correct application of TCF and limits conclusions that can be drawn. | Unique perspective on consultant stage of continuing professional development. Limitations acknowledged, including small sample size and all male participants. Not all TCs identified discussed in context of TC characteristics. Examples from data used but not always linked to TC characteristics. TCs related to ways of thinking and practicing. TCs related to uncertainty and liminality linked to professional identify formation. Technical skills identified as TCs, but also discussion of non-technical elements including uncertainty. Discussed Threshold Capabilities framework in designing a training programme to help surgeons deal with uncertainty – would require further investigation. Liminality could re-occur in experienced surgeons – nature of life-long learning in medicine related to TCs. | Novel perspective on TCF: a practical application of suprasubjectivity in medical education is through identifying, teaching and assessing TCs. Included examples of identified TCs from medical education literature – offers a new way of looking at these TCs and suggests that suprasubjectivity may underpin these transformations.   Further research needed to explore interventions that enable this to be applied to medical education and assessment in practice. |

|  | Vaughan (2016)50 | Wilkinson et al. (2016)29 | Wilkinson (2018)26 | Winter et al. (2020)27 |
| --- | --- | --- | --- | --- |
| Aims and purpose | "Are there particular work experiences for GPs that are more important than others in their development? If so, are there ways to think about these experiences which could help GPs (and their teachers) meet the challenge of practice with inherent uncertainty? This paper suggests there might be such experiences and useful ways to understand them. It offers the emergent idea of vocational thresholds as a frame around learning experiences that are transformational in nature. It suggests this may help GPs to 'know, do, and be' as practitioners negotiating uncertainty on a daily basis."(p100) | "The following analysis aims to answer two questions: (1) What influenced the junior doctors to become dementia champions? (2) What impact did being a dementia champion have on the junior doctors?"(p265) | "This article will explore the TCs crossed by junior doctors in training to become a geriatrician with a qualitative study of trainers (consultants, the educational supervisors of junior doctors), and trainees (the junior doctors)."(p65) | "A simulation day was established, based on communicating with a person with dementia in a number of scenarios... This study sets out to evaluate whether learning occurred and, if so, how this learning may happen through threshold concepts, to identify if simulation is a successful method of teaching about dementia care."(p2919,2922) |
| Country | New Zealand | United Kingdom | United Kingdom | United Kingdom |
| Medical speciality | General practice | Dementia care | Geriatric medicine | Dementia care |
| Research approach | Written accounts of learning experiences. | Semi-structured focussed group discussion; questionnaire. | Interpretive phenomenological approach: semi-structure interviews and questionnaire. | Questionnaire and field notes. |
| Participants/ population | General practitioners (GPs). | Junior doctors. | Medical educators; junior doctors including speciality trainees. | Undergraduate medical students. |
| Nature of inquiry | Creates (brings TCF into conversation with a new body of ideas; identifies experiences that may help participants cross thresholds). | Identify (names a potential TC). Discuss (discusses relation to some TC characteristics). | Identify (identifies original TCs). Discuss (discusses TCs in relation to some TC characteristics). Explains and Analysis (draws on findings from data and literature to explain why TCs address an educational issue).” | Creates (uses TCF in teaching). |
| Paper findings | “…most responses (n = 48) offered accounts focused on the development of dispositional attributes for general practice… Of the 48 responses, 30 (62.5%) focused on dispositional development learning described through informal discussions with mentors or colleagues and/or purposeful learning arrangements (eg debriefings, video analysis of their consultations)… Sixteen of the 48 responses focused on dispositional development described important learning occurring through a personal, direct experience. These were nearly all accounts of interactions with patients that revealed a dimension of patient care through the doctor-patient relationship that was new to the GP.”(p101,102) | "During the project, the junior doctor dementia champions underwent a transformative change - initiating the change to delivering hands-on care and routinely gaining detailed personal information about their dementia patients. It is likely that this is a threshold concept in geriatric medicine and, more widely, in the training of all doctors working in secondary care who are likely to be responsible for the health of older people."(p271) | “The two main themes were drawn from all three methods of analysis, and included some of the troublesome concepts. These were: an understanding of frailty / complexity in both physiology and the management and investigation of older people (termed ‘complexity of medical care’), and that of a caring persona interested in the wider wellbeing of the patient (termed ‘nurturing-care’). The troublesome areas blend into both of these broad themes.”(p68) | "The study highlights that the simulation day is successful in enhancing learning by leading to a transformative change in learner knowledge and behaviour. This learning occurs by taking learners to their perceived thresholds of capability and by challenging their thoughts and assumptions taking them to a new level of understanding."(p2925) |
| Paper conclusions/ recommendations | "A particularly fruitful approach is to take the unplanned experiences and integrate them with planned and structured opportunities for shared reflection and feedback."(p104) | No additional conclusions regarding TCF. | “The conceptualisation of these two threshold concepts of nurturing-care and complexity, places the field of geriatric medicine at the centre of a reformation of care. Junior doctors in medicine and surgery look after the most dependant population in hospital (other than the neonatal patients). They are a vulnerable group that need active care of their complex conditions and people to care about their care. To make this the norm, an expansive learning approach… is needed within the NHS as an organisation.”(p75) | “This study adds to the understanding of teaching and learning within dementia care and highlights the strength of simulation as a method to learn about communication and how learning may occur through threshold concepts.”(p2926) |
| Our findings/ appraisal | Novel approach of focussing on powerful learning experiences to identify moments of transformation and the experiences that led to them – offers valuable insight that could be used to improve training curricula design and support of continuing professional development. Used of the lens of vocational thresholds – this model requires further research. Distinction of vocational thresholds from TCs indicates variation in the interpretation of TCF. | TCF introduced in discussion and an association made between transformative change and potential TC – examples from data provided throughout the study to support the identified themes, but none were specifically described in the context of transformation. No evidence provided to support any other TC characteristic. Further research would be needed to identify if this was indeed a TC and how this could inform curricula development. | Robust approach involving both trainers and trainees. Small sample size but rich in-depth qualitative data set. Provided examples from data to support identification of themes. Did not describe “nurturing-care” TC in relation to all TC characteristics. Challenge identifying irreversibility as based on author opinion not evidence from data.  Used concept-mapping approach – appeared to be an effective way of exploring and revealing connections between concepts and also demonstrates web-like nature of TCs. TCs related to ways of thinking and practising and provided insight into the need to experience tacit phenomenon which can be TCs. Some suggestions made for how to address the identified concepts but requires further research. | Novel approach to explore TCF. Limitations acknowledged and suggestions made for future research. Good sample size. Examples from data provided to support identified themes. Focus on change in thoughts and language as evidence of TC crossing but did not explore elements related to other TC characteristics. No discussion of the potential TCs that the students may have crossed during the learning experience – need for further research. |
